# Safe Predictor-Feedback CACC with V2X-Aware Adaptive Spacing for Heterogeneous Vehicle Platoons

**DOI:** 10.3390/s26154806

**Published:** 2026-07-28

**Authors:** Jaehyeon Shin, Junhyeok An, Sungjin Lee

**Affiliations:** Department of Smart Automotive, Soonchunhyang University, 22 Soonchunhyang-ro, Sinchang-myeon, Asan-si 31538, Republic of Korea; jater@sch.ac.kr (J.S.); junhteok0227@sch.ac.kr (J.A.)

**Keywords:** cooperative adaptive cruise control, predictor feedback, string stability, V2X, vehicle platooning, safe inter-vehicle distance

## Abstract

Vehicle-to-everything (V2X)-enabled cooperative adaptive cruise control (CACC) is a key technology for improving both traffic efficiency and driving safety in vehicle-platooning scenarios. However, real-world platoons consist of heterogeneous vehicles with different actuation, computation, and mechanical delays, and communication latency also varies over time. Therefore, conventional approaches based on homogeneous vehicles and fixed-delay assumptions may fail to guarantee physical rear-end collision avoidance under severe driving conditions. This paper proposes Safe PF-CACC, a predictor-feedback-based CACC framework that integrates a V2X-aware safe inter-vehicle distance (Safe IV Distance) model with adaptive time-headway scheduling for heterogeneous vehicle platoons. The proposed Safe IV Distance is computed by considering communication latency, vehicle dynamic delays, and friction-dependent braking limits. It consists of three components: a minimum margin (MM) for low-speed and standstill conditions, a response-lag loss (RLL) induced by communication and vehicle dynamic delays, and a braking-performance limit (BPL) caused by road-friction-dependent braking capability. The resulting Safe IV Distance is converted into a dynamic effective time headway and incorporated into the predictor-feedback (PF) controller, while a filtering process is applied to suppress abrupt gain-scheduling variations. To evaluate the proposed framework, three representative CACC scenarios were considered: heterogeneous passenger-vehicle platooning, emergency vehicle platooning, and truck platooning. The simulation results show that overly short spacings without real-time delay awareness can cause collisions in high-speed and emergency driving scenarios, whereas overly conservative spacings improve safety at the cost of increased road occupancy. In the heterogeneous passenger-vehicle scenario, the proposed Safe PF-CACC reduces the maximum jerk and mean spacing by 20.6% and 49.4%, respectively, compared with the existing conservative method. In the emergency vehicle scenario, it achieved collision-free operation while reducing the maximum jerk and mean spacing by 18.6% and 53.2%, respectively. In the truck-platooning scenario, stable jerk and acceleration responses are maintained while the mean spacing is reduced by 59.6%. These results demonstrate that the proposed framework provides a practical integrated control approach for maintaining both control stability and physical safety in CACC systems under time-varying communication delays and road friction uncertainty.

## 1. Introduction

With the rapid deployment of automated driving technologies, vehicle platooning and V2X-enabled cooperative adaptive cruise control (CACC) have received increasing attention as key applications in highway driving and freight transportation. By sharing the velocity, acceleration, and control-related information of preceding vehicles, CACC enables more stable vehicle following with shorter inter-vehicle spacing than sensor-only adaptive cruise control (ACC), thereby improving both traffic efficiency and driving safety [1,2].

However, many existing platooning studies rely on simplified assumptions in which homogeneous vehicles operate under identical communication conditions and employ the same control structure. Such assumptions are difficult to satisfy in practical deployment. In real platoons, computation delays, mechanical actuation delays, and response time constants differ across vehicles owing to variations in powertrain systems, braking actuators, electronic control units, and controller implementation. In addition, V2X communication latency and reliability can vary over time owing to the wireless channel conditions, resource scheduling, and retransmission mechanisms. These heterogeneous and time-varying characteristics can degrade safe-spacing control and string stability in real driving environments [2]. Although the constant time-headway (CTH) policy is widely adopted owing to its simple implementation, it is sensitive to system delays and vehicle dynamics, which may lead to stability limitations under practical operating conditions [3,4]. Consequently, delay variability and vehicle heterogeneity can amplify disturbances in platoons and may cause rear-end collision risks, particularly under severe acceleration or braking maneuvers.

String stability is a fundamental criterion for vehicle platooning because it ensures that disturbances are not amplified as they propagate downstream along the platoon. Therefore, it has been widely used in CACC design and in the analysis of communication quality effects on platoon performance [2,3]. Recently, predictor feedback (PF)-based CACC methods have been proposed to handle heterogeneous actuation delays and provide theoretical stability and string stability guarantees under delay mismatch conditions [5,6]. However, these theoretical guarantees do not directly imply physical safety spacing. Although predictor-feedback control is effective in suppressing divergence and oscillation caused by delays, real-time generation of collision-aware target spacing requires the simultaneous consideration of total response delay, communication latency, heterogeneous vehicle response characteristics, and braking-performance differences.

To address these issues, this paper proposes an integrated Safe PF-CACC framework that incorporates a V2X-aware safe inter-vehicle distance model into the PF controller structure. The proposed Safe IV Distance is designed using three components: (i) a minimum margin (MM) for low-speed and standstill conditions, (ii) a response-lag loss (RLL) caused by communication latency and heterogeneous vehicle dynamic delays, and (iii) a braking-performance limit (BPL) that accounts for friction-dependent braking-distance differences. The computed safe distance was converted into a dynamic effective time headway, and a filtering process was applied to mitigate abrupt variations in the target spacing and control gain. By integrating real-time delay and braking-related factors into the spacing policy, the proposed framework enables adaptive gain scheduling, in which the controller automatically adjusts its feedback sensitivity according to the instantaneous risk level.

The main contributions of this paper can be summarized as follows:

- A V2X-aware adaptive PF-CACC framework based on the Safe IV Distance is proposed. The framework jointly considers real-time V2X communication latency, computation and mechanical delays, actuator response time constants, and road friction-dependent braking limits. The resulting Safe IV Distance is converted into a dynamic effective time headway and incorporated into the PF controller, allowing both the time headway and control gain to be adaptively scheduled according to the driving risk.
- A unified control structure was maintained under heterogeneous and time-varying delay conditions. The proposed formulation preserves a common feedback control form even when vehicles have different delay characteristics, thereby enabling consistent implementation for heterogeneous vehicle platoons.
- The proposed framework is quantitatively evaluated for multiple platooning scenarios. Heterogeneous passenger-vehicle platooning, emergency vehicle platooning, and truck platooning are considered, and the performance is compared with existing methods using metrics such as jerk, acceleration, time-to-collision (TTC), spacing violation, mean spacing, and string-stability ratio. Parameter guidelines are also discussed according to the vehicle type and operational purpose.

The remainder of this paper is organized as follows. Section 2 reviews related work. Section 3 presents the system model and proposed integrated control structure based on Safe IV Distance and dynamic time-headway scheduling. Section 4 quantitatively evaluates the stability and safety performance of the proposed method using simulations. Finally, Section 5 concludes the study and discusses key insights.

## 2. Related Work

### 2.1. CACC, String Stability, and Information-Flow Topologies

Vehicle platooning and cooperative adaptive cruise control (CACC) have been widely studied as enabling technologies for improving traffic throughput, fuel efficiency, and longitudinal driving safety by allowing vehicles to share motion and control-related information through V2V/V2X communication [1,7]. Compared with sensor-only adaptive cruise control (ACC), CACC can use the acceleration, velocity, and control input of preceding or leading vehicles, thereby reducing reaction delay and allowing shorter inter-vehicle spacing under appropriate stability conditions [8,9].

A fundamental requirement in vehicle platooning is string stability, which ensures that velocity, acceleration, or spacing disturbances are not amplified as they propagate downstream along the platoon [3,10,11]. Prior studies have analyzed string stability under various vehicle-following policies, controller structures, and information-flow topologies. In particular, the choice of information-flow topology, such as predecessor following, predecessor-leader following, bidirectional topology, or multiple-predecessor topology, strongly affects disturbance attenuation and scalability [12,13]. These studies provide important theoretical foundations for CACC design. However, most focus primarily on disturbance propagation and control stability, while the direct mapping from physical collision-avoidance requirements to real-time spacing and controller parameters is often not explicitly addressed.

### 2.2. Spacing Policies and Time-Headway Selection

The constant time-headway (CTH) policy is widely used in ACC and CACC because it provides a simple relationship between desired spacing and vehicle speed. Nevertheless, the minimum feasible time headway is strongly affected by actuator dynamics, communication delay, information topology, and measurement uncertainty [3,11]. Delay-based and disturbance-aware spacing policies have therefore been proposed to improve string stability under bounded disturbances and time delays [14]. Other studies have shown that V2V communication and multiple-predecessor information can reduce the required time headway and improve traffic efficiency [15].

More recent work has examined the influence of noisy or lossy V2V communication on headway selection and string stability. For example, noisy V2V communication can impose additional constraints on the minimum admissible headway, while packet losses may deteriorate disturbance attenuation and require more conservative controller settings [16,17]. These studies demonstrate that spacing policies should not be selected independently of communication quality. However, they typically focus on stability or robustness margins rather than computing a physically grounded safe distance from real-time communication latency, heterogeneous response delays, and road-friction-dependent braking capability.

### 2.3. Communication-Aware and V2X-Constrained Platoon Control

The performance of CACC is highly dependent on communication latency, packet reception rate, update interval, and network congestion. The influence of communication delay on platoon string stability has been studied in early V2V platooning research [18], and the effect of packet loss and beaconing interval on CACC performance has also been analyzed [19]. Network-aware CACC studies have further established quantitative relationships between control performance and communication requirements [2]. In addition, V2X-based platoon-control experiments have demonstrated that communication-assisted longitudinal control can improve tracking performance, but also that control performance is sensitive to wireless-channel conditions and information update quality [20].

From the communication perspective, LTE-V2X and 5G NR-V2X sidelink technologies have been investigated as key enablers of direct vehicle-to-vehicle communication [21,22]. These studies clarify the physical-layer and resource-allocation mechanisms that affect latency and reliability in vehicular networks. However, communication-level analyses are often separated from vehicle-level closed-loop control design. As a result, there remains a gap between packet-level V2X latency/reliability evaluation and the real-time adaptation of CACC spacing policies and feedback gains.

### 2.4. Delay Compensation and Predictor-Feedback CACC

Because vehicle platoons are affected by both communication delay and actuation delay, various delay-compensation methods have been proposed. Smith predictor-based CACC compensates for actuator delay by predicting future vehicle states [23]. Robust CACC designs have also considered communication delay in the space domain [24], while distributed consensus-based approaches have addressed time-varying and heterogeneous communication delays in platoons [25]. Controller synthesis methods for string-stable platoons have provided systematic design procedures under specific dynamic and information assumptions [26].

Recently, predictor-feedback (PF)-based CACC has attracted attention because it can explicitly compensate for input or actuation delays and provide rigorous stability properties. PF-CACC has been developed for CTH-based platoons with delay compensation and safety considerations [6]. It has also been extended to nonlinear vehicle dynamics with input delay [27], simultaneous compensation of actuation and communication delays in heterogeneous platoons [28], and exact predictor-feedback CACC for heterogeneous vehicles with distinct actuation delays [5]. In parallel, predictive spacing strategies based on higher-order heterogeneous vehicle models have been proposed to improve platoon control under heterogeneous dynamics [29]. Despite these advances, most PF-CACC and predictive-spacing approaches still rely on fixed or preselected headway parameters and do not explicitly embed friction-dependent braking limits and time-varying V2X latency into the internal control-gain scheduling mechanism.

Among predictor-feedback CACC studies, the works most directly related to the present paper are the robust CTH PF-CACC of Bekiaris-Liberis [6] and the exact PF-CACC for heterogeneous platoons with distinct actuation delays by Samii and Bekiaris-Liberis [5]. Bekiaris-Liberis [6] establishes rigorous robustness, safety, and string-stability properties for CTH-based PF-CACC, including positivity of spacing and speed states. This result provides a strong theoretical foundation for fixed-headway predictor-feedback CACC. However, its main formulation is based on a prescribed CTH policy and does not address a state-dependent headway that is generated online from V2X latency, heterogeneous response delays, and friction-dependent braking limits. Samii and Bekiaris-Liberis [5] further develop an exact predictor-feedback CACC framework for heterogeneous vehicles with distinct actuation delays. This method provides an exact delay-compensation mechanism for heterogeneous platoons, but it does not explicitly convert packet-level V2X latency and road-friction-dependent braking-performance differences into a real-time safe inter-vehicle distance or a scheduled spacing-error gain.

The proposed Safe PF-CACC should therefore be viewed as complementary to these PF-CACC results rather than as a replacement for them. We adopt the predictor-feedback structure to compensate vehicle-level delays, while adding a V2X-aware and friction-aware Safe IV Distance model. The computed safety distance is transformed into a dynamic effective time headway and then incorporated into the PF gain matrix. This additional scheduling mechanism allows the controller to adapt its feedback sensitivity according to the instantaneous physical risk level, but it also makes the closed-loop dynamics time-varying. For this reason, this paper additionally formulates a sufficient LPV stability condition for the scheduled controller.

### 2.5. Safety-Oriented Spacing and Physical Collision Avoidance

Physical rear-end collision avoidance has also been studied in classical car-following and modern safety-critical control. Gipps’ car-following model introduced a braking-distance-based safe-speed concept that explicitly considers the ability of a following vehicle to avoid collision under leader braking [30]. More recently, formal safety approaches such as control barrier functions (CBFs) have been used to impose safety constraints in feedback control systems [31], and the responsibility-sensitive safety (RSS) model has provided rule-based safe-distance conditions for automated driving [32]. These approaches are valuable because they explicitly address collision avoidance rather than only disturbance attenuation.

However, safety-oriented spacing models are not usually integrated with predictor-feedback CACC for heterogeneous platoons subject to both vehicle-level delays and V2X communication latency. In particular, braking-distance-based safety models often do not account for packet-level communication delay, while communication-aware CACC studies often do not include friction-dependent braking-distance differences in the feedback-control parameters. Therefore, the link between physical safe distance and real-time CACC gain scheduling remains insufficiently developed.

### 2.6. Research Gap and Positioning of This Paper

The above studies show that CACC research has progressed along several complementary directions: string-stability analysis, information-flow topology design, spacing-policy selection, communication-aware control, predictor-feedback delay compensation, and safety-critical collision avoidance. Nevertheless, these topics have largely been addressed separately. Existing string-stability and PF-CACC studies provide important delay-compensation mechanisms, but they usually do not convert real-time V2X latency and friction-dependent braking limits into an adaptive safe-spacing policy. Conversely, safety-spacing and braking-distance models address collision avoidance, but they are rarely embedded into a predictor-feedback CACC architecture for heterogeneous vehicles with distinct actuation and processing delays.

To bridge this gap, this paper proposes a Safe PF-CACC framework that computes a V2X-aware safe inter-vehicle distance in real time by jointly considering communication latency, heterogeneous processing and mechanical delays, actuator response characteristics, and road-friction-dependent braking limits. The computed safe distance is converted into a dynamic effective time headway and incorporated into the predictor-feedback controller through adaptive gain scheduling. In this way, the proposed method connects communication-level delay awareness, vehicle-level physical safety, and control-level string-stability-oriented feedback design within a unified CACC framework.

## 3. Methodology

### 3.1. Scenario Description

**Figure 1:**
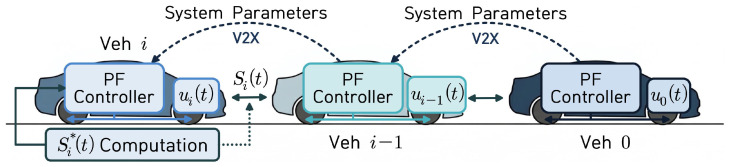
Operational Scenario of a V2X-enabled Heterogeneous Vehicle Platoon with PF Controller and Safe IV Distance.

Figure 1 illustrates the vehicle platooning system considered in this study. Once a platoon is formed, ego vehicle *i* follows its preceding vehicle i−1 with an inter-vehicle spacing $S_{i}\left(t\right)$, whereas the entire platoon follows the leading vehicle 0. To avoid rear-end collisions, each vehicle maintains its spacing based on the proposed Safe IV Distance, denoted by $S_{i}^{*}\left(t\right)$, which is updated in real-time through longitudinal acceleration control.

All vehicles in the platoon broadcast the system parameters through V2X communication. These parameters includes time-varying states, such as velocity v(t), acceleration a(t), acceleration command u(t), and spacing S(t), as well as vehicle-specific fixed parameters related to actuation, response, and braking performance. The fixed parameters are exchanged during platoon initialization, whereas time-varying states are transmitted periodically during the platoon operation. Each vehicle selectively receives the required system parameters and computes its acceleration command u(t) using its predictor-feedback (PF) controller for real-time acceleration control.

In particular, this study addresses physical factors that are not explicitly considered in conventional PF-based CACC, including inter-vehicle communication latency and degradation of braking performance caused by road friction variation. Accordingly, we define a Safe IV Distance model $S_{i}^{*}\left(t\right)$ that accounts for both communication-induced and physical delay effects and integrate it with the PF controller to construct the proposed Safe PF-CACC framework.

### 3.2. Derivation of Safe IV Distance

The proposed safe inter-vehicle distance $S_{i}^{*}\left(t\right)$ represents the minimum required spacing for preventing physically rearranged collisions, even under severe driving conditions such as sudden braking of the preceding vehicle. It incorporates the communication latency, heterogeneous vehicle response delays, and friction-dependent braking limits. The Safe IV Distance is designed using three components, namely minimum margin (MM), response-lag loss (RLL), and braking-performance limit (BPL), as follows:

$$\begin{array}{c} S_{i}^{*}\left(t\right)=\underset{\mathrm{MM}}{\underbrace{S_{0}}}+\underset{\mathrm{RLL}}{\underbrace{v_{i}\left(t\right)\cdot \max\left\{0,\Delta T_{\mathrm{RLL}}\left(t\right)\right\}}}+\underset{\mathrm{BPL}}{\underbrace{\max\left\{0,\Delta S_{\mathrm{brk}}\left(t\right)\right\}}}, \end{array}$$

where $\Delta T_{\mathrm{RLL}}\left(t\right)$ denotes the response-time difference caused by the communication latency and heterogeneous vehicle dynamics, and $\Delta S_{\mathrm{brk}}\left(t\right)$ represents the braking distance deficit between ego vehicle *i* and its preceding vehicle i−1. A detailed derivation and analysis of the MM, RLL, and BPL terms are presented in the following subsections.

#### 3.2.1. Minimum Margin (MM)

Under extreme deceleration or congested traffic conditions, the theoretical target spacing can become excessively small. In practical driving environments, sensor noise, positioning uncertainty, and small control oscillations may lead to unsafe behavior or closed-loop divergence. Therefore, a minimum spacing margin $S_{0}$ is introduced to improve the control robustness and avoid collisions under low-speed and standstill conditions. In this study, $S_{0}$ was set to 2.0m.

#### 3.2.2. Response Lag Loss (RLL)

RLL represents the additional spacing required to compensate for the difference in response times between ego vehicle *i* and its preceding vehicle i−1. Here, $D_{C,i}\left(t\right)$ is the real-time communication latency following a Gaussian distribution, formulated by the mean communication latency ${\bar{D}}_{C,i}$ and the communication-environment compensation term $\delta D_{C,i}\left(t\right)$:

$$\begin{array}{c} D_{C,i}\left(t\right)={\bar{D}}_{C,i}-\delta D_{C,i}\left(t\right). \end{array}$$

Then, the effective response time of ego vehicle *i* is modeled as the sum of the mean communication latency ${\bar{D}}_{C,i}$, communication-environment compensation term $\delta D_{C,i}\left(t\right)$, processing and mechanical delay $D_{PM,i}$, and actuator response time constant $\tau_{i}$:

$$T_{i}\left(t\right)={\bar{D}}_{C,i}-\delta D_{C,i}\left(t\right)+D_{PM,i}+\tau_{i}.$$

By contrast, the response time of the preceding vehicle i−1 is given by

$$T_{i-1}=D_{PM,i-1}+\tau_{i-1}.$$

The communication latency is included only in the response time of ego vehicle *i*, because it corresponds to the delay experienced when ego vehicle *i* receives system parameters from the preceding vehicle. The communication delay of vehicle i−1 did not directly affect the relative response calculation between the two vehicles.

The resulting response-time difference is defined as

(1) $$\begin{array}{cc} \Delta T_{RLL}\left(t\right)= & \left({\bar{D}}_{C,i}-\delta D_{C,i}\left(t\right)\right)+\left(D_{PM,i}-D_{PM,i-1}\right)\\ & +(\tau_{i}-\tau_{i-1}), \end{array}$$

(2) $$\begin{array}{cc} \mathrm{RLL} & \equiv v_{i}\left(t\right)\cdot \max\left\{0,\Delta T_{\mathrm{RLL}}\left(t\right)\right\}. \end{array}$$

#### 3.2.3. V2X Communication Delay Model

To specify the communication latency $D_{C,i}\left(t\right)$ introduced in the RLL formulation, this subsection first models the packet-level end-to-end latency in a 5G NR V2X sidelink Mode 2 environment. The average delay of deadline-satisfied packets is then used to characterize the communication latency experienced by vehicle *i*.

In this environment, platoon vehicles exchange packets to share control and safety-related information. In Mode 2, vehicles select radio resources in a distributed manner without a centralized scheduler, following a sensing-based resource selection procedure [22,33].

**Figure 2:**
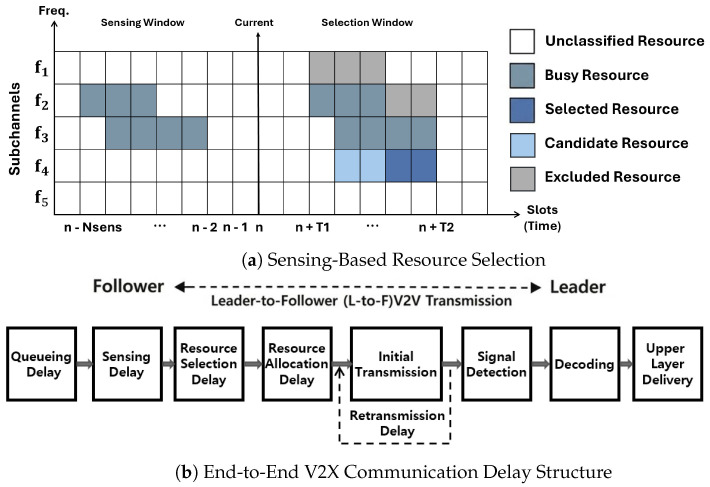
V2X Resource Selection and Delay Structure.

As shown in Figure 2a, the resource grid is represented in the time-frequency domain, where the horizontal axis denotes time slots and the vertical axis denotes sidelink subchannels. At the current slot *n*, each vehicle observes the previous sensing window, from $n-N_{\mathrm{sens}}$ to n−1, to identify resources that are already occupied or reserved by neighboring vehicles. Resources detected as busy during the sensing window are excluded from the candidate resource set because they have a high probability of collision. In addition, resources reserved by sidelink control information (SCI) are also excluded, since they are expected to be used by other vehicles in future slots.

After the sensing procedure, the vehicle evaluates the remaining resources within the future selection window, from $n+T_{1}$ to $n+T_{2}$. The unexcluded resources in this interval are regarded as candidate resources, and one of them is selected for packet transmission. The selected resource must satisfy the packet delay budget so that the generated packet can be delivered within its deadline. Thus, the sensing window is used to infer past and reserved resource occupancy, whereas the selection window determines the future transmission opportunity for the packet. This procedure follows the autonomous sensing-based resource selection operation of NR V2X sidelink Mode 2 described in 3GPP TR 38.885 [33].

After resource selection, the SCI is transmitted over the physical sidelink control channel (PSCCH) to indicate the control information required for decoding the corresponding physical sidelink shared channel (PSSCH) payload, including the resource assignment, modulation and coding scheme (MCS), and retransmission information [34,35]. In this study, PSCCH decoding failure and PSSCH data decoding failure were modeled as separate packet-delivery failure events.

Generated packets can be broadly classified into two types: periodic and aperiodic packets. Periodic packets are used to share vehicle state information, whereas aperiodic packets are generated in response to event-driven situations. The packet-generation model was configured by referring to the V2X traffic model and evaluation methodology in 3GPP TR 37.885 [36]. In the simulation, periodic traffic was configured with a packet size of 300B, generation period of 100ms, and deadline of 100ms. For aperiodic traffic, the packet size and deadline were set as 200B and 50ms, respectively. The packet inter-arrival time was modeled using an Erlang distribution with a mean of 50ms. The shape parameter was set to k=2, which yielded $\lambda_{\mathrm{ap}}=k/\left(50\;\mathrm{ms}\right)$.

Because periodic packets are generated at regular intervals, this study also considers semi-persistent scheduling (SPS) for periodic traffic. The SPS reuses previously selected resources for periodically generated packets over a certain duration while allowing resource reselection when channel congestion or resource occupancy conditions change [33]. In other words, SPS first proposes the resource to be reused for periodic traffic. If the proposed resource is no longer valid within the current selection window or has a high collision probability, an alternative resource is selected through the standard Mode 2 resource selection procedure. This abstraction allows periodic packet transmission to benefit from resource reuse while still reflecting resource reselection caused by changing channel occupancy and collision risk.

Each vehicle uses the selected resources to transmit packets to other vehicles in the platoon. In a platooning environment, the leader can transmit packets to follower vehicles, and follower vehicles can transmit packets to other vehicles within the platoon. This study focuses on packet delivery to multiple destination vehicles inside a platoon and analyzes the resulting communication latency. A packet is regarded as successfully delivered only when the required PSCCH/PSSCH decoding processes are completed and the packet is delivered to all intended destination vehicles within its deadline.

Here, *t* denotes the time index at which the platoon packets are generated and the communication latency is evaluated. In this study, the NR sidelink time axis was modeled using a slot-based structure with a numerology index $\mu_{\mathrm{NR}}=0$. This configuration corresponds to a subcarrier spacing of 15kHz, one slot per 1ms subframe, and a slot duration of $T_{\mathrm{slot}}=1\;\mathrm{ms}$ [34]. Accordingly, packet generation, resource selection, transmission, retransmission, and receiver-side processing are evaluated on a 1ms slot-level time grid.

The end-to-end communication latency of packet *p* is defined as the elapsed time from packet generation to successful delivery to all intended destination vehicles. Accordingly, the mean communication latency ${\bar{D}}_{C,i}$ is computed using only the packets that satisfy the deadline constraint as follows:

(3) $${\bar{D}}_{C,i}=\frac{\sum_{p\in P_{i}}1_{p}^{\left(\mathrm{all}\right)}D_{e2e,p}}{\sum_{p\in P_{i}}1_{p}^{\left(\mathrm{all}\right)}}.$$

Here, *p* denotes the packet index, and $P_{i}$ denotes the set of packets transmitted by vehicle *i* and is considered in the analysis. Let $D_{e2e,p}$ be the end-to-end latency of packet *p*. Because packets delivered after the deadline are less useful for safety-critical control, ${\bar{D}}_{C,i}$ is computed using only the end-to-end latencies of deadline-satisfied packets in $P_{i}$.

Here, $1_{p}^{\left(\mathrm{all}\right)}$ is an indicator function that equals one if packet *p* is successfully delivered to all destination vehicles within the deadline, and zero otherwise.

The end-to-end latency of packet *p* is given by

$$D_{e2e,p}=\left(n_{\mathrm{del},p}-n_{\mathrm{arr},p}\right)T_{\mathrm{slot}}.$$

where $n_{\mathrm{del},p}$ is the final slot index at which packet *p* is successfully delivered to all destination vehicles and $n_{\mathrm{arr},p}$ is the slot index at which packet *p* arrives at the transmission queue.

Figure 2b illustrates the delay structure from packet arrival at the queue to final delivery through resource selection, transmission, retransmission, and receiver-side processing. Accordingly, the end-to-end latency is modeled as the sum of the queuing delay, scheduling delay, transmission delay, and processing delay:

$$D_{e2e,p}=D_{q,p}+D_{s,p}+D_{tx,p}+D_{proc,p}.$$

The queuing delay $D_{q,p}$ represents the waiting time from packet arrival at the transmission queue until the packet is selected as the head-of-line (HOL) packet:

$$D_{q,p}=\left(n_{\mathrm{deq},p}-n_{\mathrm{arr},p}\right)T_{\mathrm{slot}}.$$

where $n_{\mathrm{deq},p}$ is the slot index at which packet *p* is dequeued as the HOL packet and enters the resource selection and scheduling processes.

The scheduling delay $D_{C,s}$ denotes the time required for the HOL packet to obtain transmission resources. It is modeled as the sum of the sensing delay $D_{\mathrm{sense},p}$, resource selection delay $D_{\mathrm{sel},p}$, and resource allocation delay $D_{\mathrm{alloc},p}$:

$$D_{s,p}=D_{\mathrm{sense},p}+D_{\mathrm{sel},p}+D_{\mathrm{alloc},p}.$$

Here, $D_{\mathrm{sense},p}$ is the delay associated with sensing the channel occupancy, $D_{\mathrm{sel},p}$ is the delay required to select transmission resources from the candidate set, and $D_{\mathrm{alloc},p}$ is the delay until the selected resources are reflected in the actual transmission schedule.

The transmission delay $D_{tx,p}$ includes the time required for wireless transmission and retransmission. In this study, SINR-based adaptive modulation and coding (AMC) is applied. For broadcast transmission, the modulation and coding scheme (MCS) is selected based on the lowest SINR among the destination vehicles in $D_{p}$, where $D_{p}$ denotes the destination set of packet *p* [37]. Let *d* denote a destination vehicle index, and let ${\mathrm{SINR}}_{p,d}$ be the signal-to-interference-plus-noise ratio of packet *p* at destination *d*. The required number of transmission slots $N_{tx,p}$ is determined by packet size *L* and the selected MCS [37]. The transmission delay is then expressed as:

$$D_{tx,p}=N_{tx,p}\;T_{\mathrm{slot}}+D_{r,p}.$$

where $D_{r}\left(p\right)$ denotes the retransmission delay:

$$D_{r,p}=\left(n_{\mathrm{del},p}-n_{\mathrm{end},p}^{\left(1\right)}\right)T_{\mathrm{slot}},$$

where $n_{\mathrm{end},p}^{\left(1\right)}$ is the slot index at which the first transmission attempt of packet *p* is completed.

The processing delay $D_{proc,p}$ represents the receiver-side processing latency and is modeled as

$$D_{proc,p}=D_{det,p}+D_{dec,p}+D_{deliver,p}.$$

Here, $D_{det,p}$ denotes the delay for signal detection and synchronization; $D_{dec,p}$ denotes the delay for demodulation and decoding; and $D_{deliver,p}$ denotes the delay for delivering the decoded packet to the upper layer [34,35].

#### 3.2.4. Braking Performance Limit (BPL)

The braking-performance limit compensates for the mismatch in braking distance caused by differences in the road friction coefficient μ, which may vary owing to weather conditions, road surface states, or tire wear. It is assumed that the friction coefficient μ remains constant within a unit time step but may vary across different time steps. The frictional force $F_{\mathrm{fric}}$ generated during vehicle braking is given as the product of the friction coefficient μ, the vehicle mass *m*, and the gravitational acceleration *g*, yielding $F_{\mathrm{fric}}=\mu mg$.

Therefore, the work done by the frictional force can be expressed as the product of the force $F_{\mathrm{fric}}$ and the braking distance $S_{\mathrm{brk},i}\left(t\right)$, which is $\mu mgS_{\mathrm{brk},i}\left(t\right)$. According to the principle of energy conservation, this is equivalent to the kinetic energy of the vehicle. That is, the following relationship holds, where both sides share the same unit of energy:

$$\mu mgS_{\mathrm{brk},i}\left(t\right)=\frac{1}{2}mv_{i}{\left(t\right)}^{2}.$$

Rearranging this equation with respect to the braking distance $S_{\mathrm{brk},i}\left(t\right)$ mathematically eliminates the mass variable *m* from both sides. Consequently, the final braking distance is derived as follows, and its unit is explicitly yielded in meters:

$$S_{\mathrm{brk},i}\left(t\right)=\frac{v_{i}{\left(t\right)}^{2}}{2\mu g}.$$

To avoid collisions under extreme deceleration scenarios, an additional clearance margin equal to the difference in braking distances between the ego vehicle *i* and the preceding vehicle i−1 is required. The difference in braking distances between the two vehicles is defined as:

(4) $$\begin{array}{c} \Delta S_{\mathrm{brk}}\left(t\right)=\frac{v_{i}{\left(t\right)}^{2}}{2\mu_{i}g}-\frac{v_{i-1}{\left(t\right)}^{2}}{2\mu_{i-1}g}. \end{array}$$

If the braking distance of the ego vehicle *i* is shorter than that of the preceding vehicle, the calculated value becomes negative, implying that no additional braking margin is required. Therefore, the final BPL term is defined with a lower bound of zero as follows:

(5) $$\begin{array}{c} \mathrm{BPL}\equiv \max\left\{0,\Delta S_{\mathrm{brk}}\left(t\right)\right\}. \end{array}$$

### 3.3. Safe PF-CACC

**Figure 3:**
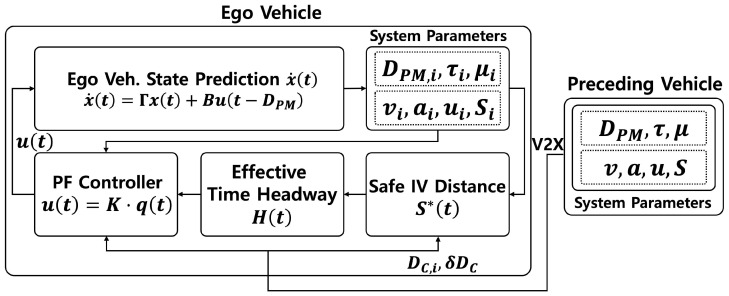
Safe PF-CACC: Overall Control Architecture integrating the Safe IV Distance Model and PF Controller.

Figure 3 shows the overall control architecture of the proposed Safe PF-CACC. The ego vehicle receives the system parameters of the preceding vehicle via V2X communication. These parameters include time-varying states, such as velocity, acceleration, control input, and inter-vehicle spacing, as well as vehicle-specific parameters, such as processing and mechanical delay, actuator time constant, and road-friction coefficient. Through the V2X communication process, the ego vehicle obtains the communication latency $D_{C,i}$ and corresponding compensation term $\delta D_{C}$. The proposed Safe IV Distance model then computes $S_{i}^{*}\left(t\right)$ by jointly using the received information from the preceding vehicle and the ego vehicle’s own parameters, while accounting for communication latency, heterogeneous vehicle dynamics, and braking-performance limits.

The computed $S_{i}^{*}\left(t\right)$ is converted into the effective time headway $H_{i}\left(t\right)$, which is incorporated into the gain matrix $K_{i}$ of the PF controller. This allows the controller to adaptively adjust its feedback sensitivity according to the physical risk level and V2X communication conditions. The PF controller generates the final acceleration command $u_{i}\left(t\right)$, and the ego vehicle state prediction block updates the vehicle states while considering processing and mechanical actuation delays. Consequently, the proposed framework enables safe-spacing control and stable string platooning, even under heterogeneous vehicle characteristics and time-varying communication latency.

#### 3.3.1. Derivation of the Effective Time Headway

This subsection describes how the proposed Safe IV Distance $S_{i}^{*}\left(t\right)$ is converted into the effective time headway $H_{i}\left(t\right)$ used in the gain matrix $K_{i}$ of the PF controller. First, the time headway $h_{i}\left(t\right)$ is obtained by normalizing $S_{i}^{*}\left(t\right)$ with the current speed of ego vehicle *i* as follows:

(6) $$h_{i}\left(t\right)=\frac{S_{i}^{*}\left(t\right)}{\max(v_{i}\left(t\right),\epsilon )}.$$

Here, ϵ is a positive regularization constant that prevents $h_{i}\left(t\right)$ from diverging at extremely low speeds. The resulting headway can be bounded by the design upper bound $h_{\max}$, depending on the operating conditions.

Because $S_{i}^{*}\left(t\right)$ includes nonlinear operations and discontinuous positive-part functions, abrupt variations can be induced by sensor noise or severe braking events. To mitigate such variations, a first-order low-pass filter (LPF) is applied to $h_{i}\left(t\right)$:

$$\tau_{f}{\dot{h}}_{i}^{*}\left(t\right)+h_{i}^{*}\left(t\right)=h_{i}\left(t\right),$$

where $\tau_{f}$ denotes the filter time constant. The LPF limits the rate of variation of the scheduled control gain. In this study, the latency introduced by the LPF is assumed to be marginal compared with the overall execution time of the real-time control loop. In addition, to prevent the filtered headway from falling below a physically admissible minimum time headway $h_{\min}$, the final effective time headway $H_{i}\left(t\right)$ is defined as:

(7) $$H_{i}\left(t\right)=\max\left\{h_{i}^{*}\left(t\right),h_{\min}\right\}.$$

Thus, $H_{i}\left(t\right)$ remains lower-bounded by $h_{\min}$, which prevents an excessive feedback gain under low-risk or low-speed conditions.

#### 3.3.2. Application of Predictor Feedback

This subsection explains how the effective time headway $H_{i}\left(t\right)$ in (7) is integrated into the PF controller to generate the final acceleration command $u_{i}\left(t\right)$. The PF controller is divided into two cases based on the relationship between the processing and mechanical delay of ego vehicle *i* and that of its preceding vehicle i−1. According to [5], when the processing and mechanical delay of ego vehicle *i* is not greater than that of the preceding vehicle, that is, $D_{PM,i}\leq D_{PM,i-1}$, the future state of ego vehicle *i* over the delay interval $D_{PM,i}$ can be predicted using the information of the preceding vehicle. This case is referred to as a standalone case.

By contrast, when the processing and mechanical delay of ego vehicle *i* is greater than that of the preceding vehicle, that is, $D_{PM,i}>D_{PM,i-1}$, the prediction requires additional information from multiple upstream vehicles beyond vehicle i−1. This case is referred to as the extended case, in which an augmented state vector $x_{i}\left(t\right)$ is constructed to fully predict the future state of the ego vehicle *i*.

In both cases, the acceleration command is expressed using the predicted state vector $q_{i}\left(t\right)$ and gain matrix $K_{i}$:

(8) $$u_{i}\left(t\right)=K_{i}\cdot q_{i}\left(t\right).$$

Here, $u_{i}\left(t\right)$ denotes the final acceleration command applied to the actuator.

##### Standalone Case ($D_{PM,i}\leq D_{PM,i-1}$)

In the standalone case, the current state vector of ego vehicle *i* is defined using the inter-vehicle spacing $S_{i}\left(t\right)$, the velocity $v_{i}\left(t\right)$, the acceleration $a_{i}\left(t\right)$, and the velocity and acceleration of the preceding vehicle, $v_{i-1}\left(t\right)$ and $a_{i-1}\left(t\right)$:

$$x_{i}\left(t\right)={\left[\begin{array}{ccccc} S_{i}\left(t\right) & v_{i}\left(t\right) & v_{i-1}\left(t\right) & a_{i}\left(t\right) & a_{i-1}\left(t\right) \end{array}\right]}^{T}.$$

Then, the state derivative ${\dot{x}}_{i}\left(t\right)$ governed by the state-space model can be defined as:

(9) $$\begin{array}{c} {\dot{x}}_{i}\left(t\right)=\Gamma_{i}x_{i}\left(t\right)+B_{i}u_{i}\left(t-D_{PM,i}\right). \end{array}$$

In (9), $\Gamma_{i}$ is the system matrix describing the longitudinal vehicle dynamics, and $B_{i}$ is the control matrix that restricts the effect of the acceleration command solely to the acceleration derivative within the state variables $x_{i}\left(t\right)$.

To explicitly represent the contributions of ego vehicle *i* and preceding vehicle i−1, the system matrix and control matrices are given by

$$\begin{array}{cc} \Gamma_{i} & =\left[\begin{array}{ccccc} 0 & -1 & 1 & 0 & 0\\ 0 & 0 & 0 & 1 & 0\\ 0 & 0 & 0 & 0 & 1\\ 0 & 0 & 0 & -\frac{1}{\tau_{i}} & 0\\ 0 & 0 & 0 & 0 & -\frac{1}{\tau_{i-1}} \end{array}\right],\\ B_{i}^{i} & ={\left[\begin{array}{ccccc} 0 & 0 & 0 & \frac{1}{\tau_{i}} & 0 \end{array}\right]}^{T},B_{i}^{i-1}={\left[\begin{array}{ccccc} 0 & 0 & 0 & 0 & \frac{1}{\tau_{i-1}} \end{array}\right]}^{T}. \end{array}$$

In the stand-alone case, the state derivative ${\dot{x}}_{i}\left(t\right)$ incorporates the elements of the preceding vehicle i−1 through the five-dimensional state vector $x_{i}\left(t\right)$. Accordingly, the state derivative of the ego vehicle is formulated as

$${\dot{x}}_{i}\left(t\right)=\Gamma_{i}x_{i}\left(t\right)+B_{i}^{i}u_{i}\left(t-D_{PM,i}\right)+B_{i}^{i-1}u_{i-1}\left(t-D_{PM,i-1}\right).$$

Similar to $x_{i}\left(t\right)$, the predicted state vector $q_{i}\left(t\right)$ in the standalone case consists of five components associated with ego vehicle *i* and the preceding vehicle i−1. The control parameters $\alpha_{i}$, $b_{i}$, and $c_{i}$ of the control gain matrix $K_{i}$ are fixed design constants for maintaining the platoon stability. Specifically, $\alpha_{i}$, $b_{i}$, and $c_{i}$ determine the compensation values for spacing error, relative velocity, and acceleration difference, respectively. The predicted state vector $q_{i}\left(t\right)$ and control gain matrix $K_{i}$ are defined as follows:

$$\begin{array}{cc} q_{i}\left(t\right) & =[S_{i}\left(t+D_{PM,i}\right)\;\;v_{i}\left(t+D_{PM,i}\right)\;\;v_{i-1}\left(t+D_{PM,i}\right),\\ & \;\;a_{i}\left(t+D_{PM,i}\right)\;\;a_{i-1}\left(t+D_{PM,i}\right){]}^{T},\\ K_{i} & =\left[\begin{array}{ccccc} \frac{\tau_{i}\alpha_{i}}{H_{i}\left(t\right)} & -\tau_{i}\left(\alpha_{i}+b_{i}\right) & \tau_{i}b_{i} & -\tau_{i}c_{i} & \tau_{i}c_{i} \end{array}\right],\\ u_{i}\left(t\right) & =\frac{\tau_{i}\alpha_{i}}{H_{i}\left(t\right)}S_{i}\left(t+D_{PM,i}\right)\\ \; & -\tau_{i}\left(\alpha_{i}+b_{i}\right)v_{i}\left(t+D_{PM,i}\right)+\tau_{i}b_{i}v_{i-1}\left(t+D_{PM,i}\right)\\ \; & -\tau_{i}c_{i}a_{i}\left(t+D_{PM,i}\right)+\tau_{i}c_{i}a_{i-1}\left(t+D_{PM,i}\right). \end{array}$$

##### Extended Case ($D_{PM,i}>D_{PM,i-1}$)

Unlike the standalone case, the extended case requires additional upstream vehicle information because the shorter actuation delay of the preceding vehicle produces a prediction gap. In this case, the control decision of vehicle i−1 may depend on the state of vehicle i−2 or even further upstream vehicles. Therefore, the state components of vehicle i−2, such as $S_{i-1}$, $v_{i-2}$, and $a_{i-2}$, or those of the additional preceding vehicles, must be included in the prediction.

Accordingly, the current state vector of ego vehicle *i* is redefined as an augmented state vector $x_{i}\left(t\right)$ with dimension eight or higher, depending on the number of upstream vehicles included in the prediction. The augmented state derivative ${\dot{x}}_{i}\left(t\right)$ is expressed as follows:

(10) $${\dot{x}}_{i}\left(t\right)=\Gamma_{i}x_{i}\left(t\right)+\sum_{j=i-N_{p}}^{i}B_{i}^{j}u_{j}\left(t-D_{PM,j}\right).$$

Here, $x_{i}\left(t\right)$, ${\dot{x}}_{i}\left(t\right)$, $\Gamma_{i}$, and $B_{i}^{j}$ denote the augmented state vector, augmented state derivative, augmented system matrix, and the corresponding control matrices, respectively. The parameter $N_{p}$ represents the number of preceding vehicles required for prediction. Index *j* denotes each vehicle involved in the prediction, ranging from the furthest upstream vehicle $i-N_{p}$ to ego vehicle *i*.

For instance, when reflecting the information up to vehicle i−2 ($N_{p}=2$), the predicted state vector $q_{i}^{i}\left(t\right)$ and the explicit control gain matrix $K_{i}$ are formulated in an 8-dimensional space as follows:

(11) $$\begin{array}{cc} q_{i}^{i}\left(t\right) & =[S_{i}\left(t+D_{PM,i}\right)\;\;S_{i-1}\left(t+D_{PM,i}\right)\;\;v_{i}\left(t+D_{PM,i}\right),\\ & \;v_{i-1}\left(t+D_{PM,i}\right)\;\;v_{i-2}\left(t+D_{PM,i}\right)\;\;a_{i}\left(t+D_{PM,i}\right),\\ \; & \;a_{i-1}\left(t+D_{PM,i}\right)\;\;a_{i-2}\left(t+D_{PM,i}\right){]}^{T}, \end{array}$$

(12) $$\begin{array}{cc} K_{i} & =\left[\begin{array}{cccccccc} \frac{\tau_{i}\alpha_{i}}{H_{i}\left(t\right)} & 0 & -\tau_{i}\left(\alpha_{i}+b_{i}\right) & \tau_{i}b_{i} & 0 & -\tau_{i}c_{i} & \tau_{i}c_{i} & 0 \end{array}\right]. \end{array}$$

Even though the extended case scales up in dimensionality and complexity as more upstream vehicles are incorporated, the structure of the final feedback control law is perfectly isomorphic to that of the standalone case. This structural consistency arises from the sparse matrix property of the augmented control gain matrix $K_{i}$, which assigns zero components to the intermediate state variables not directly involved in the control law (e.g., $S_{i-1}$, $v_{i-2}$, and $a_{i-2}$). Since these intermediate variables are naturally eliminated during the computation, the final acceleration command $u_{i}\left(t\right)$ for the extended case is identically derived as the simple inner product of $q_{i}^{i}\left(t\right)$ and $K_{i}$, exactly matching the form of Equation (8).

#### 3.3.3. Analysis of Adaptive Gain Scheduling

After the PF controller generates the final acceleration command $u_{i}\left(t\right)$, the state derivative ${\dot{x}}_{i}\left(t\right)$ is updated, leading to the next vehicle state $x_{i}\left(t\right)$ in the closed loop. In the proposed Safe PF-CACC framework, the fixed time-headway parameter used in conventional PF-CACC is replaced by the dynamic effective time headway $H_{i}\left(t\right)$. When the physical risk level increases owing to the sudden braking of the preceding vehicle or increased communication latency, the Safe IV Distance $S_{i}^{*}\left(t\right)$ increases, which in turn increases $H_{i}\left(t\right)$.

Because $H_{i}\left(t\right)$ appears in the denominator of the spacing error gain term in $K_{i}$, a larger $H_{i}\left(t\right)$ value reduces the corresponding feedback gain. This results in more conservative and smoother behavior. Conversely, when the communication condition is reliable and the physical risk level is low, $H_{i}\left(t\right)$ decreases, thereby increasing the feedback gain and enabling a more responsive vehicle following. Thus, the proposed controller realizes adaptive gain scheduling, where the feedback sensitivity is adjusted in real time according to the external driving conditions and physical risk level.

### 3.4. Sufficient Stability Condition for Adaptive Gain Scheduling

The proposed Safe PF-CACC replaces the fixed time-headway parameter of conventional PF-CACC with the dynamic effective time headway $H_{i}\left(t\right)$. Since $H_{i}\left(t\right)$ appears in the denominator of the spacing-error gain, the resulting closed-loop system becomes time-varying and state-dependent. Therefore, the stability property of a fixed-headway PF-CACC cannot be directly invoked. This subsection provides a sufficient stability condition for the proposed scheduled controller.

First, the effective time headway is constrained to an admissible compact interval:

(13) $$H_{i}\left(t\right)\in \left[h_{i,\min},h_{i,\max}\right],\;0<h_{i,\min}<h_{i,\max}<\infty .$$

This condition is satisfied by the lower bound in the effective-headway definition and by imposing an upper design bound on the scheduled headway. The bound $h_{i,\max}$ is selected to cover the maximum expected Safe IV Distance under the prescribed operating domain. Equivalently, define the scheduling variable

(14) $$\theta_{i}\left(t\right)=\frac{1}{H_{i}\left(t\right)},\;\theta_{i}\left(t\right)\in \left[{\underline{\theta }}_{i},{\bar{\theta }}_{i}\right],$$

where ${\underline{\theta }}_{i}=1/h_{i,\max}$ and ${\bar{\theta }}_{i}=1/h_{i,\min}$. Since the spacing-error gain is proportional to $1/H_{i}\left(t\right)$, the gain-scheduled controller can be represented using $\theta_{i}\left(t\right)$.

After applying the predictor transformation, the compensated pairwise closed-loop dynamics of vehicle *i* can be written in the following LPV form:

(15) $${\dot{\xi }}_{i}\left(t\right)=A_{i}\left(\theta_{i}\left(t\right)\right)\xi_{i}\left(t\right)+B_{i}w_{i-1}\left(t\right),\;y_{i}\left(t\right)=C_{i}\xi_{i}\left(t\right)+D_{i}w_{i-1}\left(t\right),$$

where $\xi_{i}\left(t\right)$ denotes the compensated local tracking-error state, $w_{i-1}\left(t\right)$ denotes the disturbance or motion input propagated from the preceding vehicle, and $y_{i}\left(t\right)$ denotes the output used for string-stability evaluation, such as velocity or acceleration. The scheduled system matrix is affine in $\theta_{i}\left(t\right)$:

(16) $$A_{i}\left(\theta_{i}\right)=A_{i,0}+\theta_{i}A_{i,1}.$$

The matrices $A_{i,0}$, $A_{i,1}$, $B_{i}$, $C_{i}$, and $D_{i}$ are obtained from the predictor-compensated vehicle dynamics and the PF gain matrix.

**Theorem 1.**  *Consider the predictor-compensated LPV system above. Suppose that, for each vehicle i, there exist a symmetric positive-definite matrix $P_{i}=P_{i}^{⊤}≻0$, a scalar $\gamma_{i}\leq 1$, and a scalar $\varepsilon_{i}>0$ such that the following bounded-real LMI holds for both vertices $\theta_{i}\in \left\{{\underline{\theta }}_{i},{\bar{\theta }}_{i}\right\}$:*

(17) $$\left[\begin{array}{cc} A_{i}{\left(\theta_{i}\right)}^{⊤}P_{i}+P_{i}A_{i}\left(\theta_{i}\right)+C_{i}^{⊤}C_{i}+\varepsilon_{i}I\; & P_{i}B_{i}+C_{i}^{⊤}D_{i}\\ B_{i}^{⊤}P_{i}+D_{i}^{⊤}C_{i}\; & D_{i}^{⊤}D_{i}-\gamma_{i}^{2}I \end{array}\right]⪯0.$$

*Then, for any measurable scheduled headway satisfying $H_{i}\left(t\right)\in \left[h_{i,\min},h_{i,\max}\right]$, the local closed-loop system is uniformly stable and satisfies the induced $L_{2}$ gain bound*

(18) $${∥y_{i}∥}_{2}\leq \gamma_{i}{∥w_{i-1}∥}_{2}+\sqrt{\lambda_{\max}\left(P_{i}\right)}∥\xi_{i}\left(0\right)∥.$$

*If the initial tracking errors are zero and $\prod_{i=1}^{N}\gamma_{i}\leq 1$, the platoon is $L_{2}$ string stable over the admissible scheduling range.*

**Proof.**  Consider the common quadratic Lyapunov function

(19) $$V_{i}\left(\xi_{i}\right)=\xi_{i}^{⊤}P_{i}\xi_{i}.$$

For a fixed value of $\theta_{i}$, differentiating $V_{i}$ along the LPV dynamics yields

(20) $${\dot{V}}_{i}=\xi_{i}^{⊤}\left(A_{i}{\left(\theta_{i}\right)}^{⊤}P_{i}+P_{i}A_{i}\left(\theta_{i}\right)\right)\xi_{i}+2\xi_{i}^{⊤}P_{i}B_{i}w_{i-1}.$$

Using the LMI in (17), we obtain

(21) $${\dot{V}}_{i}+y_{i}^{⊤}y_{i}-\gamma_{i}^{2}w_{i-1}^{⊤}w_{i-1}\leq -\varepsilon_{i}\xi_{i}^{⊤}\xi_{i}.$$

Because $A_{i}\left(\theta_{i}\right)$ is affine in $\theta_{i}$, and because the LMI is convex in $\theta_{i}$, feasibility at the two vertices ${\underline{\theta }}_{i}$ and ${\bar{\theta }}_{i}$ implies feasibility for all $\theta_{i}\left(t\right)\in \left[{\underline{\theta }}_{i},{\bar{\theta }}_{i}\right]$. Therefore, the inequality holds for any admissible time-varying scheduling trajectory. Integrating both sides from 0 to *T* gives

(22) $$\int_{0}^{T}y_{i}^{⊤}y_{i}dt\leq \gamma_{i}^{2}\int_{0}^{T}w_{i-1}^{⊤}w_{i-1}dt+V_{i}\left(0\right),$$

which proves the stated induced $L_{2}$ gain bound. If $\xi_{i}\left(0\right)=0$ and the product of the local induced gains along the platoon is not larger than unity, disturbances are not amplified downstream. Hence, the platoon is $L_{2}$ string stable. □

The theorem provides a sufficient rather than necessary condition. It also clarifies that the proposed method does not claim unconditional string stability under arbitrary unbounded delay or friction variations. Instead, the guarantee holds when the V2X latency, vehicle response delays, and friction-dependent braking terms induce a scheduled headway within the admissible interval. Variations outside this operating domain should be handled by a fallback safety mode, such as emergency braking or platoon disengagement.

## 4. Results

To evaluate the effectiveness of the proposed Safe PF-CACC, three representative platooning scenarios, denoted by S1, S2, and S3, were considered. Under the same operating conditions, the proposed method was compared with representative existing approaches, including those of Ploeg [26], Chen [29], and Samii [5]. A comparison was performed using the safety and stability verification metrics listed in Table 1.

- S1: Baseline platooning of heterogeneous passenger vehicles with seven vehicles.
- S2: Emergency platooning of special-purpose vehicles with five vehicles.
- S3: Freight-truck platooning with seven vehicles.

We first analyze the communication-latency performance, which is a key input to the proposed Safe IV Distance model. The three platooning scenarios are then evaluated based on the communication-latency results.

### 4.1. Communication-Latency Evaluation

Before analyzing the communication-latency results, this subsection presents the communication simulation environment and the main parameters used in the evaluation. Based on the packet-level NR V2X sidelink Mode 2 communication-delay model defined in Section 3.2.3, this study employed a custom Python (version 3.12.13)-based packet-level system-level simulator. The simulator abstracts the NR V2X sidelink Mode 2 sensing-based resource-selection procedure and the V2X packet-delivery process at the system level. Specifically, the autonomous resource-selection and sidelink operation of NR V2X Mode 2 were configured by referring to 3GPP TR 38.885 [33], while the V2X traffic-generation and evaluation methodology were configured by referring to 3GPP TR 37.885 [36]. The modulation order, target code rate, and spectral efficiency used for MCS selection were configured by referring to the MCS table structure in 3GPP TS 38.214 [37]. The PSCCH/PSSCH transmission and decoding processes were abstracted at the system level based on the NR sidelink physical-channel and channel-coding procedures defined in 3GPP TS 38.211 [34] and TS 38.212 [35].

Monte Carlo simulations were conducted using $n_{\mathrm{drop}}=1000$ independent repetitions for each communication case. In each repetition, the initial vehicle positions, packet-generation times, Mode 2 resource-selection outcomes, and channel-shadowing components were independently generated.

The basic wireless communication environment, road configuration, vehicle-density conditions, and Monte Carlo evaluation parameters used in the simulations are summarized in Table 2.

The frequency-domain sidelink resource-pool configuration and the physical-resource parameters used for packet transmission are summarized in Table 3. These parameters define the amount of radio resources available for sidelink transmission and provide the basis for modeling resource occupancy and resource-pool congestion.

Table 3 summarizes the frequency-domain sidelink resource-pool configuration and the physical-resource parameters used for PSCCH and PSSCH transmission. The resource-pool bandwidth, number of subchannels, subchannel size, and PRB allocation define the amount of radio resources available for simultaneous sidelink transmissions. In addition, the OFDM-symbol, DMRS, and transmission-layer configurations determine the physical-resource structure used for sidelink control-information and packet-data transmission. Because the available sidelink resources are finite, these parameters provide the basis for modeling resource occupancy, competition among transmitting vehicles, and resource-pool congestion in the packet-level simulation.

The propagation and antenna models used to calculate the received power of each vehicle-to-vehicle link, including the LOS/NLOSv pathloss, shadow fading, antenna pattern, and vehicle-blockage loss, are summarized in Table 4.

The links between platoon vehicles were classified as either line-of-sight (LOS) or non-line-of-sight due to vehicle blockage (NLOSv) based on the Highway evaluation methodology in 3GPP TR 37.885 [36]. A link was classified as LOS when no other platoon vehicle was located between the transmitting and receiving vehicles. When another platoon vehicle was located between the transmitter and receiver and blocked the direct propagation path, the corresponding link was classified as NLOSv.

The LOS pathloss $PL_{\mathrm{LOS}}\left(d\right)$ was calculated using the effective link distance $d_{\mathrm{eff}}$ between the transmitting and receiving vehicles and the carrier frequency $f_{c}$. In the pathloss expression, $d_{\mathrm{eff}}$ is expressed in meters, while $f_{c}$ is expressed in GHz. The effective link distance was lower-bounded by 2m to prevent the pathloss from decreasing to an unrealistically low value at very short inter-vehicle distances. The distance-dependent term $21\log_{10}\left(d_{\mathrm{eff}}\right)$ represents the increase in propagation loss with link distance, whereas the frequency-dependent term $20\log_{10}\left(f_{c}\right)$ accounts for the effect of carrier frequency on the pathloss.

For an NLOSv link, the additional vehicle-blockage loss $L_{\mathrm{block}}$ was added to the LOS pathloss. All platoon vehicles were configured with the same antenna height of 3m. Therefore, the NLOSv Case-3 model specified in 3GPP TR 37.885 [36] was applied because the transmitting, receiving, and blocking vehicles had the same antenna height.

The NLOSv Case-3 vehicle-blockage loss $L_{\mathrm{block}}$ was generated from a Gaussian distribution with a distance-dependent mean and a standard deviation of 4dB. If the generated value was negative, the vehicle-blockage loss was limited to 0dB to prevent a nonphysical increase in received power due to vehicle blockage.

Each platoon vehicle was equipped with a single antenna at a height of 3m, and the antenna-element pattern for a 6-GHz Type-3 Vehicle UE defined in Option 1 of 3GPP TR 37.885 [36] was applied. The vertical antenna pattern $A_{E,V}\left(\theta \right)$ represents antenna-gain attenuation as a function of the zenith angle θ, with an attenuation of 0dB at $\theta =90^{\circ }$. The horizontal antenna pattern $A_{E,H}\left(\phi \right)$ represents antenna-gain attenuation as a function of the azimuth angle ϕ, with an attenuation of 0dB in the antenna reference direction at $\phi =0^{\circ }$.

The combined antenna-element pattern $A_{E}\left(\theta ,\phi \right)$ was obtained by combining the vertical and horizontal antenna patterns. The directional antenna gain G(θ,ϕ) was then calculated by adding the combined antenna-pattern value to the maximum antenna-element gain of $G_{\max}=3\;\mathrm{dBi}$.

The final received power was calculated using the transmit power, directional gains of the transmitting and receiving antennas, link-state-dependent pathloss, and shadow-fading component as follows:

(23) $$\begin{array}{cc} P_{\mathrm{rx}}= & P_{\mathrm{tx}}+G_{\mathrm{tx}}\left(\theta_{\mathrm{tx}},\phi_{\mathrm{tx}}\right)+G_{\mathrm{rx}}\left(\theta_{\mathrm{rx}},\phi_{\mathrm{rx}}\right)\\ \; & -PL_{\mathrm{LOS}}\left(d\right)-I_{\mathrm{NLOSv}}L_{\mathrm{block}}+SF. \end{array}$$

In (23), $P_{\mathrm{tx}}$ denotes the transmit power; $G_{\mathrm{tx}}$ and $G_{\mathrm{rx}}$ denote the directional gains of the transmitting and receiving antennas, respectively; $PL_{\mathrm{LOS}}\left(d\right)$ denotes the basic LOS pathloss; $L_{\mathrm{block}}$ denotes the additional NLOSv Case-3 vehicle-blockage loss; and SF denotes the shadow-fading component. The link-state indicator $I_{\mathrm{NLOSv}}$ is defined as

(24) $$I_{\mathrm{NLOSv}}=\left\{\begin{array}{cc} 0, & \mathrm{LOS},\\ 1, & \mathrm{NLOSv}. \end{array}\right.$$

Accordingly, only the basic LOS pathloss and shadow-fading component were applied to an LOS link, whereas the additional vehicle-blockage loss $L_{\mathrm{block}}$ was included for an NLOSv link.

The time-domain resource-selection, congestion-control, retransmission, and packet-processing parameters used to model NR V2X sidelink Mode 2 scheduling and communication latency are summarized in Table 5.

Table 5 summarizes the main time-domain resource-selection, packet-scheduling, congestion-control, retransmission, and processing-delay parameters used in the NR V2X sidelink Mode 2 simulation. The sensing-window and selection-window parameters define the observation interval for identifying occupied resources and the future time interval in which transmission resources are selected. The candidate-resource selection fraction, CBR-related parameters, and SPS congestion threshold are used to represent resource availability and resource-pool congestion. In addition, the transmission-queue capacity, SPS reselection counter, retransmission parameters, and packet-processing delays determine the packet scheduling, resource reuse, retransmission, and receiver-side processing behavior. These parameters collectively determine the resource-acquisition process and the packet-level end-to-end communication latency evaluated in this study.

The communication-environment and vehicle-density parameters, sidelink resource-pool configuration, propagation and antenna models, and Mode 2 resource-selection and scheduling parameters summarized in Table 2, Table 3, Table 4 and Table 5 were used to apply the packet-level communication-delay model described in Section 3.2.3 to the Monte Carlo simulations.

Four representative communication cases were considered: (300m,4followers), (300m,5followers), (50m,4followers), and (50m,5followers). The 300m spacing case corresponds to a low-density condition with 3.3333veh/km/lane, 20 vehicles within a 1km road segment, and 12 vehicles within the communication coverage interval. The 50m spacing case corresponds to a high-density condition with 20.0000veh/km/lane, 120 vehicles within a 1km road segment, and 72 vehicles within the communication coverage interval. The number of followers denotes the number of transmitting follower vehicles in the target platoon. Thus, the transmitting vehicles consisted of one leader and either four or five transmitting followers.

The MCS was selected based on the representative SINR at the packet-transmission time. The modulation order, target code rate, and spectral efficiency used for SINR-based link abstraction were configured according to the MCS table defined in 3GPP TS 38.214 [37]. Packet success was determined by jointly considering the selected MCS, PSCCH/PSSCH decoding results, and deadline satisfaction, while the PSCCH/PSSCH transmission and decoding procedures were abstracted according to 3GPP TS 38.211 and TS 38.212 [34,35].

**Figure 4:**
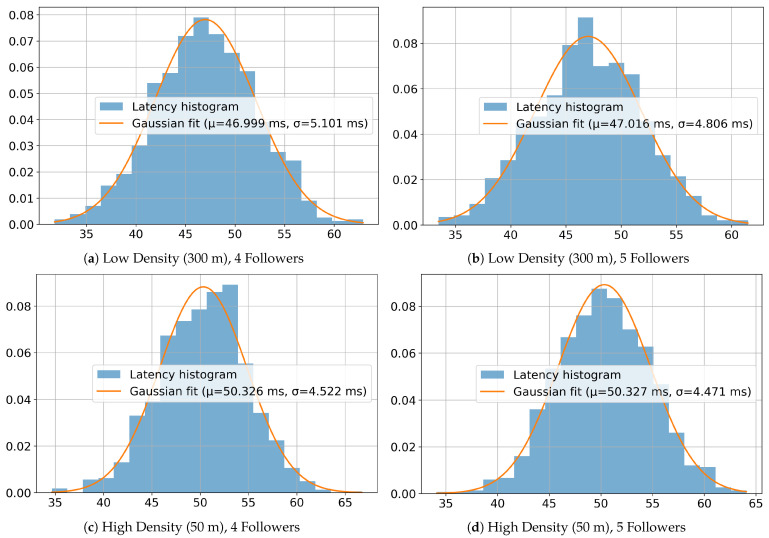
Latency Distribution with Gaussian Fitting under Low- and High-Density Conditions.

Figure 4 presents the end-to-end latency distributions obtained for the four representative communication cases. The simulation results show that the average communication latency ranged from approximately 47.0 to 50.3ms.

First, the effect of vehicle density was evaluated by comparing the low-density 300m case with the high-density 50m case. For four transmitting followers, the average latency increased from 46.999ms to 50.326ms. For five transmitting followers, the average latency increased from 47.016ms to 50.327ms. Reducing the inter-vehicle spacing from 300m to 50m increased the number of vehicles located within the communication coverage interval from 12 to 72. Consequently, more vehicles simultaneously shared the same finite sidelink resource pool, resulting in higher resource occupancy and stronger mutual interference. These changes were accompanied by a shift of the overall communication-latency distribution toward larger values under the high-density condition.

Next, the effect of the number of transmitting followers was investigated under the same vehicle-density condition. In the low-density scenario, increasing the number of transmitting followers from four to five changed the average latency only slightly, from 46.999ms to 47.016ms. Similarly, in the high-density scenario, the average latency remained almost unchanged, varying from 50.326ms to 50.327ms. These results indicate that, under the considered simulation conditions, the vehicle density had a substantially greater impact on communication latency than increasing the number of transmitting follower vehicles by one.

The standard deviation of the communication latency was approximately 5.101ms for the 300m, 4-follower case, 4.806ms for the 300m, 5-follower case, 4.522ms for the 50m, 4-follower case, and 4.471ms for the 50m, 5-follower case. Although the average latency increased under the high-density condition, the latency variation remained similar across all scenarios. Therefore, increasing vehicle density mainly shifted the center of the latency distribution rather than significantly increasing its dispersion.

Because the latency distributions in Figure 4 alone are insufficient to characterize communication reliability, the packet delivery ratio (PDR), packet error ratio (PER), and average SINR were additionally evaluated for each communication scenario. The PER was calculated as PER=1−PDR. Table 6 summarizes the corresponding PDR, PER, and average SINR results.

As shown in Table 6, the PDR was approximately 95.4% in the low-density 300m scenarios and decreased to approximately 91.7–92.1% in the high-density 50m scenarios. Correspondingly, the PER increased from approximately 4.5% to approximately 7.9–8.3%. The average SINR also decreased from approximately 43.5–43.7dB to approximately 36.8–37.1dB. These results indicate that increasing vehicle density degraded communication reliability while simultaneously reducing the received signal quality.

Overall, increasing vehicle density consistently affected the communication latency, PDR, PER, and SINR. Reducing the inter-vehicle spacing from 300m to 50m substantially increased the number of vehicles simultaneously sharing the finite resources of the 20MHz sidelink resource pool. The resulting increases in resource occupancy and mutual interference were reflected in the lower average SINR, reduced PDR, increased PER, and the shift of the latency distribution toward larger values under the high-density condition.

To further investigate the composition of the communication latency, the end-to-end delay was decomposed into its constituent components. The values reported below were obtained from a representative scenario in which only the platoon leader transmitted packets. The average delay components measured by the simulator were $D_{q,p}=0.531\;\mathrm{ms}$, $D_{\mathrm{sense},p}=1.000\;\mathrm{ms}$, $D_{\mathrm{sel},p}=0.500\;\mathrm{ms}$, $D_{\mathrm{alloc},p}=41.938\;\mathrm{ms}$, $D_{\mathrm{tx},p}=1.000\;\mathrm{ms}$, $D_{r,p}=0.089\;\mathrm{ms}$, $D_{\det,p}=0.200\;\mathrm{ms}$, $D_{\mathrm{dec},p}=1.100\;\mathrm{ms}$, and $D_{\mathrm{deliver},p}=0.500\;\mathrm{ms}$. Among these components, the resource-allocation delay $D_{\mathrm{alloc},p}$ accounted for the largest proportion of the total end-to-end latency. The queueing delay, retransmission delay, and receiver-side processing delays contributed comparatively smaller portions.

Based on the communication-performance evaluation presented above, the packet-level communication delay adopted in the subsequent CACC simulations was set to a nominal value of 50ms. This value represents the communication latency observed under the high-density conditions and was selected as a representative delay for evaluating the CACC performance. To reflect time-varying communication-delay fluctuations, Gaussian random noise with a standard deviation of 3ms was added to the nominal delay assigned to each vehicle. The delay variation was limited to ±10ms to avoid unrealistic outliers while preserving stochastic latency fluctuations.

### 4.2. Three Platooning Scenarios

#### 4.2.1. Experimental Setup

To simulate a realistic vehicle platooning environment, this simulation eliminates the simplistic assumption of constant communication delay and introduces a time-varying communication delay model. To achieve this, the stochastic distribution characteristics of the latency derived from the 5G NR V2X Monte Carlo simulation in Section 4.1 are directly coupled with the vehicle dynamics control loop based on the results in Figure 4.

Commercial vehicles have physical acceleration and deceleration limits owing to powertrain constraints, vehicle mass, and road-friction conditions [38]. Therefore, realistic acceleration limits were applied to reflect the vehicle-dynamic characteristics. The maximum acceleration values were set to $3.5\;m/s^{2}$ for sedans, $3.2\;m/s^{2}$ for SUVs, $3.0\;m/s^{2}$ for buses, and $2.5\;m/s^{2}$ for trucks. Furthermore, realistic and variable communication latency can induce high-frequency fluctuations in the effective time headway $H_{i}\left(t\right)$, which determines the control gains. Therefore, to suppress these high-frequency fluctuations while satisfying the stability condition in Section 3.4, the LPF time constant $\tau_{f}$ is set to 2.0s in this driving scenario.

The processing and mechanical delay $D_{PM,i}$ is configured based on the vehicle type: 80ms for sedans, 90ms for SUVs, 100ms for buses, and 110ms for trucks. Crucially, subtle differences are introduced to the $D_{PM,i}$ values even within the same vehicle category. This ensures that the platoon naturally comprises a mixture of both the standalone case and the extended case. Specifically, S1 and S3, which consist of similar vehicles, predominantly feature the standalone case ($D_{i}\leq D_{i-1}$), although the extended case ($D_{i}>D_{i-1}$) also naturally coexists due to slight delay variations. Additionally, this extended case can be observed in the S2 emergency scenario owing to the delay differences between distinct vehicle models, such as V1 and V2. The actuator time constant τ is adjusted according to the vehicle characteristics, with nominal values of approximately 0.3 for passenger vehicles and 0.5 for trucks.

**Figure 5:**
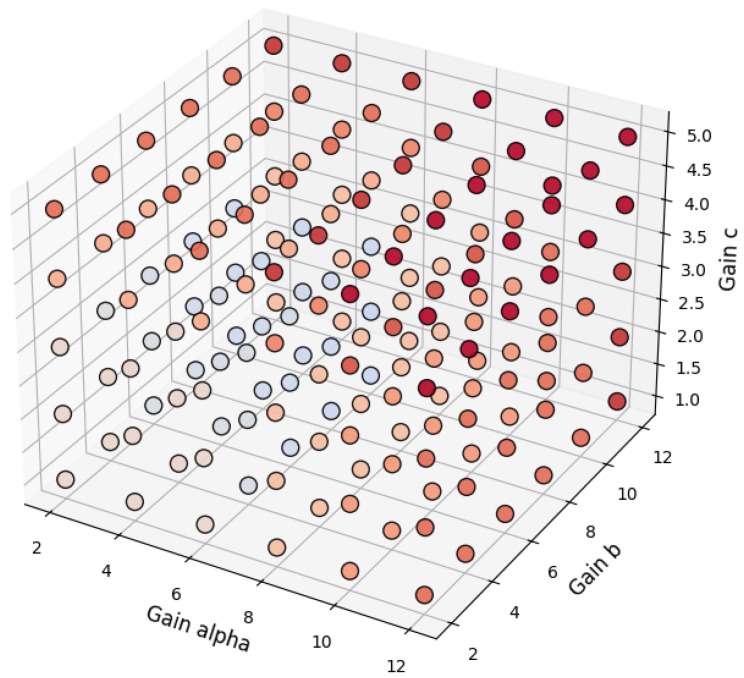
3D Parameter Space: Feasible and Unrealistic High Gain Region.

Additionally, the control parameters α, *b*, and *c* are selected by considering the trade-off between control stability and ride comfort based on the conditions of each vehicle. To accomplish this, a 3D sensitivity analysis is performed by mapping α, *b*, and *c* onto the x, y, and z axes of a three-dimensional space, respectively, as illustrated in Figure 5.

This analysis reflects real physical constraints such as jerk error limits, road-friction deceleration thresholds, and communication variables. The analysis results are integrated with the string stability values and visualized with a gradient, where the stable region is represented in blue and the unstable region in red. Accordingly, the control parameters for each vehicle applied in this scenario are selected within the blue feasible region of the graph.

Based on this environment incorporating variable communication delays and physical limitations, three driving scenarios (S1, S2, S3) are configured as shown in Table 7:

In S1, the heterogeneous passenger-vehicle platooning scenario, the platoon consists of a total of seven vehicles, with the lowest road friction coefficient assigned to vehicle V5 located at the rear of the formation. For V1–V4 and V6, the parameters are set to α=6, b=10, c=2, and $h_{\min}=0.5\;s$, while for V5, which features the low friction coefficient, α=3, b=8, and c=2 are applied. The leading vehicle, vehicle V0, is modeled as a human-driven vehicle that induces acceleration and deceleration within a speed range of approximately 20–25m/s, corresponding to about 90km/h. The following vehicles are assigned appropriate τ and μ values corresponding to passenger sedans.

In S2, the emergency platooning scenario, the control parameters α, *b*, and *c* are selected by jointly considering ride comfort, represented by jerk, and tracking performance, represented by control stability, under emergency driving conditions. The leading vehicle *L* is modeled as a police vehicle that performs aggressive maneuvers within a speed range of approximately 24–42m/s, corresponding to about 150km/h, with τ=0.25 and μ=1.0. V1 is configured as an SUV emergency rescue vehicle with τ=0.3, μ=1.0, α=4, b=8, c=2, and $h_{\min}=0.49\;s$. The relatively large $h_{\min}$ value of the emergency rescue vehicle (V1) is applied to suppress excessive jerk, considering its medical transport purpose, and to attenuate shockwaves caused by the aggressive maneuvers of the leader. V2 and V4 are modeled as large fire trucks with τ=0.5, μ=0.9, α=4, b=8, c=3, and $h_{\min}=0.35\;s$. V3 is modeled as a medium fire truck with τ=0.4, μ=1.0, α=7, b=10, c=3, and $h_{\min}=0.35\;s$.

In S3, the truck platooning scenario, the platoon consists of seven trucks operating within a speed range of approximately 20–33.3m/s, corresponding to approximately 120km/h. This range is selected to reflect realistic high-speed freight-truck operation by imposing lower acceleration and maximum-speed conditions than in S2, considering the physical characteristics of the leading truck. The leader *L* and vehicles V1–V6 are all configured with τ=0.5, μ=1.0, α=4, b=8, and c=3; however, V1 is set with $h_{\min}=0.5\;s$, whereas V2–V6 are set with $h_{\min}=0.33\;s$. The larger $h_{\min}=0.5\;s$ of V1 serves to partially absorb abrupt leader maneuvers and stabilize the motion of the following trucks. In addition, each truck features a slightly different $D_{PM,i}$ value, reflecting both the standalone case and the extended case.

#### 4.2.2. Results and Discussion

Table 8 reports the results for each scenario using the safety and stability verification metrics defined in Table 1. The proposed Safe PF-CACC is compared with the methods of Ploeg [26], Chen [29], and Samii [5]. In each metric, the values are reported in the order of Ploeg, Chen, Samii, and Safe PF-CACC. Based on the results in Table 8, safety is analyzed as the primary evaluation criterion, followed by a comparative performance analysis of the controllers in terms of inter-vehicle spacing efficiency and ride comfort.

First, evaluating safety in the S2 scenario reveals that Ploeg and Samii fail to appropriately handle the braking and latency limits under the extreme conditions of S2. Consequently, they exhibit unstable control performance compared to Chen and the proposed Safe PF-CACC. Conversely, comparing the spacing efficiency of the two collision-free controllers in the S1 and S3 scenarios demonstrates that Chen maintains an average inter-vehicle spacing approximately twice as wide as that of the proposed Safe PF-CACC. Although Chen yields marginally superior ride comfort metrics in some cases, such as S3, this is an artifact of the excessively large inter-vehicle spacing. Given the overarching goal of platooning efficiency, it cannot be considered practically superior. Furthermore, in the emergency driving conditions of S2, the proposed Safe PF-CACC yields a lower average maximum jerk than Chen, which persistently maintains wide spacing. In conclusion, when applying comprehensive evaluation criteria encompassing safety, efficiency, and ride comfort, the proposed Safe PF-CACC is confirmed to be the most robust compared to Ploeg, Chen, and Samii. A detailed causal and quantitative analysis for each scenario is provided below.

##### Safety Analysis and Collision Mechanisms Under Extreme Conditions (S2)

**Figure 6:**
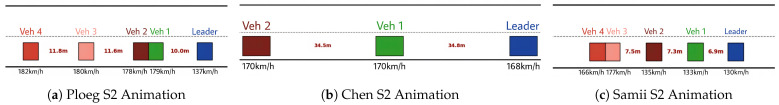
Ploeg, Chen, and Samii S2 Animation.

S2 constitutes a severe testing environment characterized by abrupt deceleration and disparate braking limits among heterogeneous vehicles (e.g., degraded braking capability of a heavy fire truck). As indicated in Table 8, Ploeg and Samii fail to maintain string stability, resulting in rear-end collisions. These moments of impact can be verified through the animation illustrated in Figure 6. In Ploeg, the fire truck V2 rear-ends the preceding rescue vehicle V1 at approximately t=23.6s as the leader decelerates, and an additional collision involves V4. Although the initial spacing before braking is not excessively tight, the accident occurs because the controller is fundamentally limited by its inability to dynamically adapt to the differing friction coefficients and physical braking capabilities among heterogeneous vehicles. Ploeg’s $H_{\infty }$ controller [26], which utilizes fixed control parameters, cannot attenuate and absorb the amplified shockwave propagating through the platoon, ultimately failing to minimize tracking errors and exhausting the clearance margin. This structural flaw in Ploeg is corroborated not only in S2 but also by the unstable string stability ratio of 1.1041 in the S3 truck scenario. Similarly, Samii incurs an accident between fire trucks V3 and V4 at approximately t=24.2s during the leader’s sudden deceleration. Because Samii’s controller is designed under the linear assumption of a constant communication delay, it can maintain tight spacing and establish a stable platoon under ideal environments where latency remains invariant. However, in environments like S2 where communication delay varies in real time and physical braking capacities are mismatched, attempting to maintain tight spacing with a fixed time headway fails to physically compensate for dynamic delay phenomena. This accumulates prediction errors, thereby leading to rear-end collisions. Furthermore, while Chen’s PSS method [29] avoids collisions and appears to offer higher ride comfort and stability compared to Ploeg and Samii, it inherently maintains the widest inter-vehicle distance among the three benchmark methods. In contrast, the proposed Safe PF-CACC achieves a 18.63% reduction in the average maximum jerk and a 53.18% reduction in mean spacing compared to Chen in the S2 scenario.

**Figure 7:**
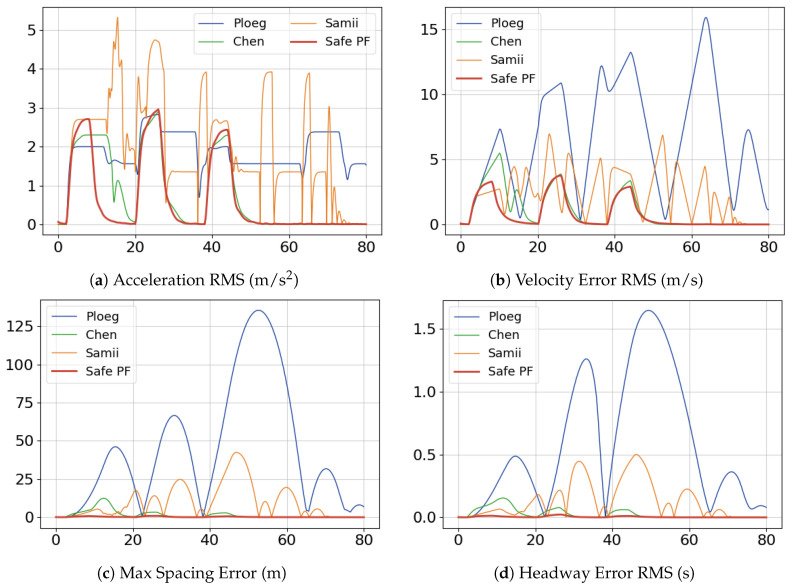
Comparative Performance of Scenario 2 (S2): Emergency Vehicle Platooning.

To comprehensively evaluate the behavior of the entire platoon (V1–V4) and validate these observations, Figure 7 illustrates the comparative platooning performance of each controller using aggregate metrics. Specifically, the root-mean-square (RMS) values are utilized for (a) acceleration, (b) velocity tracking error, and (d) time-headway error. For (c) spacing error, which is directly related to collision avoidance, the maximum absolute error ($L_{\infty }$-norm) is applied. For these metrics, values closer to zero with minimal fluctuations indicate superior ride comfort, tracking performance, and safety. As visualized in Figure 7, the Ploeg and Samii methods exhibit severe error amplification, resulting in the aforementioned collisions, while the Chen method avoids extreme error propagation, the proposed Safe PF-CACC suppresses fluctuations across all metrics, proving that it maintains the most stable and superior control flow under extreme conditions.

##### Efficiency and Ride Comfort Analysis (S1 and S3)

As summarized in Table 8, Samii and Ploeg exhibit performance metrics comparable to those of the Safe PF-CACC in the S1 and S3 scenarios. However, due to the critical safety defects exposed in the S2 analysis, they cannot serve as viable alternatives for ensuring safety in real-world road environments. Additionally, Chen exhibits practical limitations because the vehicles maintain an excessively wide inter-vehicle distance. This phenomenon is identically observed across all three scenarios, stemming from the inherent structural conservatism of Chen’s method. To aggressively suppress communication disturbances and longitudinal tracking errors, Chen employs a fixed, large baseline safety distance and a wide prediction time window. Consequently, when real-time latencies or minor velocity fluctuations occur, the controller generates overly aggressive deceleration commands (over-damping) to prevent system error propagation. Although this avoids collisions, it forces the controller to open unnecessary gaps between vehicles, rendering the primary objective of platooning—maximizing traffic efficiency and road capacity—ineffective. In contrast, the proposed Safe PF-CACC demonstrates a 20.63% reduction in maximum jerk and a 49.37% reduction in mean spacing compared to Chen in S1, alongside a 59.63% reduction in mean spacing in S3.

**Figure 8:**
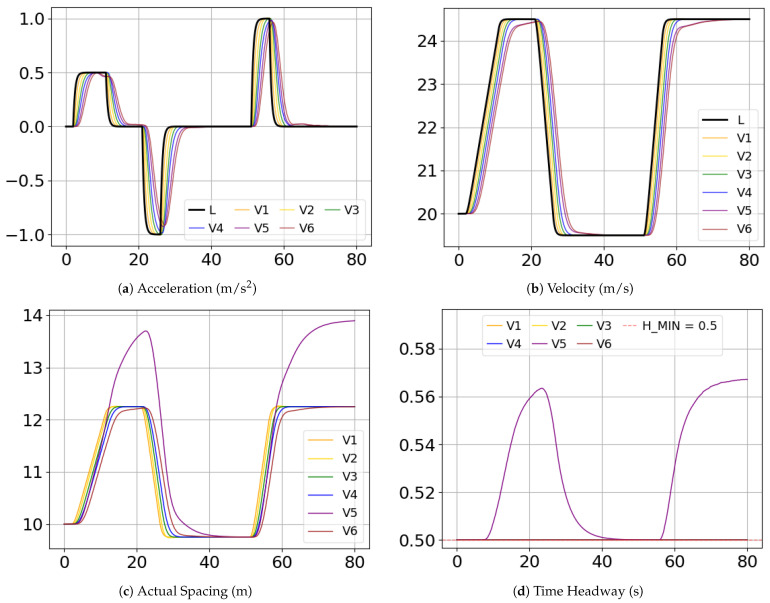
Safety Performance of Scenario 1 (S1): Heterogeneous Vehicle Platooning.

Figure 8 independently illustrates the driving trajectories of the platooned vehicles under the Safe PF-CACC in S1. As observed in (a) and (b), the following vehicles smoothly track the leader’s maneuvers. In particular, the adaptive gain-scheduling effect of the Safe PF-CACC is evident in (c) actual spacing and (d) time headway, while standard vehicles (V1–V4 and V6) drive tightly by maintaining the lower bound of $h_{\min}=0.5\;s$, vehicle V5, which features a low friction coefficient, autonomously recognizes its physical braking performance limit (BPL) through the $S_{i}^{*}\left(t\right)$ computation and dynamically scales up its time headway. Consequently, it is confirmed that V5 in S1 proactively maintains an additional clearance of approximately 2m relative to the other vehicles.

**Figure 9:**
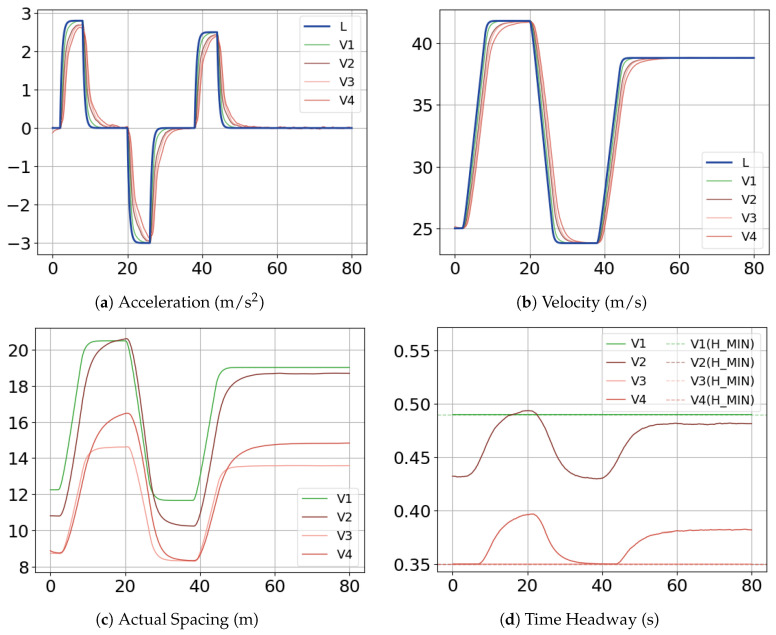
Safety Performance of Scenario 2 (S2): Emergency Vehicle Platooning.

Figure 9 displays the resulting performance curves of the Safe PF-CACC under the S2 emergency platooning scenario. Amidst extreme acceleration and deceleration responses shown in (a) and (b), the adaptive control characteristics of the proposed method are visible in (c) actual spacing and (d) time headway. As specified in Section 4.2.1 and Table 7, the extended case ($D_{\mathrm{PM},i}>D_{\mathrm{PM},i-1}$) is applied to vehicles V2 and V4 in Figure 9. Specifically, the delay of the preceding vehicle V1 is 90ms while its follower V2 is 130ms, and the preceding vehicle V3 is 110ms while its follower V4 is 130ms. Because the delay of the ego vehicle exceeds that of the preceding vehicle in these pairs, V2 and V4 are controlled using the augmented gain matrix defined in Equation (10). The simulation results demonstrate that these vehicles maintain stable responses alongside the standalone case vehicles. Notably, in (d), V2 does not merely remain at the minimum lower bound of $h_{\min}=0.37\;s$; instead, via the $S_{i}^{*}\left(t\right)$ computation, it actively increases $H_{i}\left(t\right)$ in segments with a high risk of collision, thereby yielding a high safety margin. Similarly, for V4, the time headway spikes upward during specific sharp deceleration segments, deviating from the linear trajectory of $h_{\min}$. This dynamic headway scheduling is immediately reflected as an extension of the inter-vehicle spacing in (c). Even under extreme scenarios, the Safe PF-CACC secures safety by expanding the spacing only during critical moments of danger, while returning to a tight formation during normal intervals, thereby simultaneously achieving both physical safety and spatial traffic efficiency.

**Figure 10:**
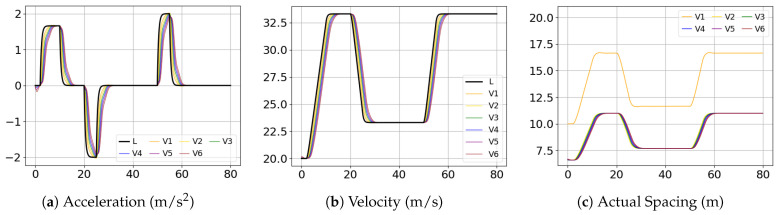
Safety Performance of Scenario 3 (S3): Truck Platooning.

Figure 10 presents the performance results of the Safe PF-CACC in the S3 truck platooning scenario, while the platooned vehicles smoothly track the leading vehicle within the trucks’ physical limitations as shown in the acceleration and velocity responses of (a) and (b), a strategic behavior of vehicle V1 is observed in (c) actual spacing. To pre-emptively absorb sequential sudden-braking shockwaves that could otherwise propagate to the subsequent vehicles due to the leader’s abrupt maneuvers, V1 proactively establishes a wide safety margin. With the disturbances neutralized through V1, the remaining following trucks (V2–V6) maintain tight, uniform intervals stably, thereby executing efficient platoon operations.

In conclusion, the proposed Safe PF-CACC controller demonstrates its superiority by expanding the inter-vehicle spacing to prevent collisions while maintaining the platoon formation under extreme situations like S2, while simultaneously securing inter-vehicle efficiency and ride comfort during normal platooning in S1 and S3.

## 5. Conclusions

This paper proposed Safe PF-CACC, which integrates a Safe IV Distance model with predictor-feedback control for heterogeneous CACC systems with communication and mechanical delays. The proposed model accounts for V2X latency, vehicle response delay, and road friction-dependent braking limits, and converts the resulting safe distance into an effective time headway for adaptive gain scheduling. Simulation results in heterogeneous passenger-vehicle, emergency vehicle, and truck platooning scenarios show that Safe PF-CACC maintains collision-free and string-stable platoon behavior while reducing excessive spacing. Compared with existing methods, the proposed approach better balances safety, ride comfort, and road-occupancy efficiency under realistic delay and vehicle-heterogeneity conditions.

## 6. Discussion

### 6.1. Practical Friction Estimation and Robustness

Regarding the practical implementation of the Braking Performance Limit (BPL) formulated in Section 3.2.4, the real-time friction coefficient can be measured using vehicle-mounted hardware sensors such as the Vaisala MD30 (Vaisala Oyj, Vantaa, Finland) [39] and is periodically updated via V2X communication. However, raw sensor measurements may contain transient high-frequency noise or errors due to temporary road irregularities. To address this, a moving-average method is applied to the sensor data for refinement. By updating μ periodically based on the average of measurements accumulated over a specific time window, transient outliers are filtered out, and a stable μ is continuously provided for the BPL computation.

### 6.2. Sensitivity Analysis for the LPF Time Constant

The LPF time constant $\tau_{f}=2.0\;s$, applied across all scenarios, was determined through a sensitivity analysis in the emergency braking scenario (S2), which serves as an extreme condition suitable for observing system variations. To conservatively evaluate the platoon performance, Max Jerk and Min TTC were extracted as the worst-case values within the platoon, while Mean Spacing was calculated as the average. As shown in Table 9, the system operated stably without collisions across all configurations. Without the LPF ($\tau_{f}=0.0$), noise resulted in a Max Jerk of $3.58\;m/s^{3}$; applying the LPF reduced this value to $2.78\;m/s^{3}$. Specifically, at $\tau_{f}=2.0$, the system secured relatively high safety (Min TTC of 8.38 s) while achieving the shortest Mean Spacing of 14.48 m. Therefore, $\tau_{f}=2.0$ was selected as the experimental parameter.

### 6.3. Fallback Strategy and Platoon Re-Engagement Conditions

The fallback strategy and platoon re-engagement conditions for handling exceptional driving scenarios outside the Linear Parameter-Varying (LPV) stability domain derived in Theorem 1 are defined as follows. First, the transition to the fallback mode is immediately activated if any of the boundary violations are met: the effective time headway exceeding the upper bound ($H_{i}\left(t\right)>h_{i,\max}$) due to V2X communication outages or road friction degradation; the required time headway violating the lower bound ($h_{i}\left(t\right)<h_{i,\min}$) due to abrupt preceding vehicle deceleration; or the absolute inter-vehicle spacing falling below a critical collision threshold (e.g., $S_{i}\left(t\right)<2\;m$). Depending on the system state, the vehicle subsequently executes conventional Adaptive Cruise Control (ACC) degradation, manual takeover, or Autonomous Emergency Braking (AEB).

Conversely, to re-engage the platooning mode from the fallback state, complete driving stability must be restored. Specifically, the system switches back to the V2X-enabled CACC platoon control if all of the following conditions are simultaneously satisfied: the V2X communication latency recovers to the nominal range, the transient disturbances from the preceding vehicle are resolved, and the effective time headway $H_{i}\left(t\right)$ stabilizes within the theoretical operational domain ($\left[h_{i,\min},h_{i,\max}\right]$).

## Figures and Tables

**Table 1:** Safety Performance Verification Metrics.

Metric	Description and Equation
Max Jerk ($J_{max}$) ↓	Metric for ride comfort and shockwave attenuation. $J_{max}=\max\left|J_{i}\left(t\right)\right|=\max{\dot{a}}_{i}$, where $J_{i}$: jerk, ${\dot{a}}_{i}$: accel. derivative
Max Accel ($A_{max}$) ↓	Maximum acceleration. $A_{max}=\max\left|a_{i}\left(t\right)\right|$
Min TTC ($TTC_{min}$) ↑	Minimum Time-To-Collision when $v_{i}>v_{i-1}$. $TTC_{min}=\min\left(\frac{S_{i}\left(t\right)}{v_{i}\left(t\right)-v_{i-1}\left(t\right)}\right)$
Mean Space ($S_{mean}$) ↓	Average inter-vehicle distance. $S_{mean}=\frac{1}{T}\sum_{t=1}^{T}S_{i}\left(t\right)$
String Stability Ratio (SSR) ↓	Disturbance attenuation metric: $SSR=\frac{\max|a_{N}\left(t\right)|}{\max|a_{0}\left(t\right)|}\leq 1$, where $a_{0}$: leader, $a_{N}$: last veh. accel

**Table 2:** Communication Environment and Vehicle-Density Configuration.

Parameter	Value
Carrier frequency	6GHz
Subcarrier spacing	15kHz
Slot duration	1ms
Transmit power	13dBm/subchannel
Receiver noise figure	9dB
Vehicle speed	140km/h
Communication coverage radius	300m
Communication coverage interval	600m
Road length	4000m
Number of lanes	6
Platoon size	6 vehicles
Inter-vehicle spacing per lane	300m or 50m
Vehicles in 1km	20 or 120
Vehicles in communication coverage	12 or 72
Target transmitting followers	4 or 5
Target transmitting vehicles	5 or 6
Simulation duration per drop	30s
Number of Monte Carlo drops	1000

**Table 3:** Sidelink Resource-Pool Configuration.

Parameter	Value
Sidelink resource-pool bandwidth	20MHz
Number of subchannels	10
Nominal subchannel bandwidth	2MHz
Subchannel size	11PRBs
PSCCH allocation per subchannel	2PRBs
PSSCH allocation per subchannel	9PRBs
Subcarriers per PRB	12
Subcarrier spacing	15kHz
OFDM symbols per slot	14
DMRS symbols per slot	2
DMRS REs per PRB per DMRS symbol	6
Number of transmission layers	1
SCI power boost	3dB

**Table 4:** Pathloss, Shadowing, and Antenna Configuration.

Parameter	Equation or Value
LOS pathloss	$PL_{\mathrm{LOS}}\left(d\right)=32.4+21\log_{10}\left(d_{\mathrm{eff}}\right)+20\log_{10}\left(f_{c}\right)$
NLOSv pathloss	$PL_{\mathrm{NLOSv}}\left(d\right)=PL_{\mathrm{LOS}}\left(d\right)+L_{\mathrm{block}}$
Effective link distance	$d_{\mathrm{eff}}=\max\left(d,2\;m\right)$
Shadowing standard deviation	$\sigma_{\mathrm{SF}}=3\;\mathrm{dB}$
Antenna height	$h_{\mathrm{ant}}=3\;m$
Maximum antenna-element gain	$G_{\max}=3\;\mathrm{dBi}$
Vertical antenna pattern	$A_{E,V}\left(\theta \right)=-\min\left\{12{\left(\frac{\theta -90^{\circ }}{90^{\circ }}\right)}^{2},20\right\}$
Horizontal antenna pattern	$A_{E,H}\left(\phi \right)=-\min\left\{12{\left(\frac{\phi }{120^{\circ }}\right)}^{2},20\right\}$
Combined antenna pattern	$A_{E}\left(\theta ,\phi \right)=-\min\left\{-\left[A_{E,V}\left(\theta \right)+A_{E,H}\left(\phi \right)\right],20\right\}$
Directional antenna gain	$G\left(\theta ,\phi \right)=G_{\max}+A_{E}\left(\theta ,\phi \right)$
NLOSv Case-3	$L_{\mathrm{block}}=\max\left\{0,N\left(5+\max\left(0,15\log_{10}\left(d\right)-41\right),4^{2}\right)\right\}$

**Table 5:** Mode 2 Resource-Selection and Scheduling Parameters.

Parameter	Value
Sensing window duration	1000ms
Minimum selection offset, $T_{1}$	1ms
Periodic selection window	100ms
Aperiodic selection window	50ms
Candidate-resource selection fraction	20%
CBR observation window	100ms
CBR congestion threshold	0.7
Transmission queue capacity	10 packets
Initial SPS reselection counter	10
SPS congestion threshold	0.8
Maximum retransmissions	2
Retransmission interval	3ms
Resource selection delay, $D_{\mathrm{sel},p}$	0.5ms
Detection delay, $D_{\det,p}$	0.2ms
Delivery delay, $D_{\mathrm{deliver},p}$	0.5ms
Decoding delay, $D_{\mathrm{dec},p}$	0.2ms + MCS/packet-size term

**Table 6:** PDR, PER, and SINR for Representative Communication Cases.

Scenario	PDR	PER	SINR [dB]
Low density, 300m, 4 followers	0.955144	0.044856	43.739
Low density, 300m, 5 followers	0.954354	0.045646	43.475
High density, 50m, 4 followers	0.921385	0.078615	37.079
High density, 50m, 5 followers	0.917334	0.082666	36.849

**Table 7:** Simulation Parameters for Each Platooning Scenario.

Scenario	Vehicle	Vehicle Type	α	*b*	*c*	$h_{\min}$ (s)	τ (s)	μ	$D_{\mathrm{PM}}$ (s)
S1	L	Sedan (Human)	-	-	-	-	0.30	1.00	0.08
	V1	Sedan	6	10	2	0.50	0.30	1.00	0.08
	V2	Sedan	6	10	2	0.50	0.30	0.95	0.08
	V3	Sedan	6	10	2	0.50	0.30	0.90	0.08
	V4	Sedan	6	10	2	0.50	0.30	1.00	0.08
	V5	Sedan (Low μ)	3	8	2	0.50	0.30	0.50	0.08
	V6	Sedan	6	10	2	0.50	0.30	1.00	0.08
S2	L	Police Car	-	-	-	-	0.25	1.00	0.08
	V1	SUV Ambulance	4	8	2	0.49	0.30	1.00	0.09
	V2	Large Fire Truck	4	8	3	0.35	0.50	0.90	0.13
	V3	Medium Fire Truck	7	10	3	0.35	0.40	1.00	0.11
	V4	Large Fire Truck	4	8	3	0.35	0.50	0.90	0.13
S3	L	Leader Truck	-	-	-	-	0.50	1.00	0.11
	V1	Freight Truck	4	8	3	0.50	0.50	1.00	0.12
	V2	Freight Truck	4	8	3	0.33	0.50	1.00	0.10
	V3	Freight Truck	4	8	3	0.33	0.50	1.00	0.11
	V4	Freight Truck	4	8	3	0.33	0.50	1.00	0.13
	V5	Freight Truck	4	8	3	0.33	0.50	1.00	0.12
	V6	Freight Truck	4	8	3	0.33	0.50	1.00	0.11

**Table 8:** Safety Performance of Scenarios S1, S2, S3.

Veh	Max Jerk (m/s^3^)				Max Accel (m/s^2^)				Min. TTC (sec)				Mean Space (m)			
	Ploeg	Chen	Samii	Safe PF	Ploeg	Chen	Samii	Safe PF	Ploeg	Chen	Samii	Safe PF	Ploeg	Chen	Samii	Safe PF
S1. Heterogeneous Vehicle Platooning																
V1	1.19	1.19	0.82	0.90	1.01	0.99	1.01	1.00	22.48	46.41	20.68	20.79	13.10	22.08	11.11	11.10
V2	0.74	0.81	0.59	0.57	0.99	0.97	1.02	1.00	25.79	44.84	20.96	20.94	13.09	22.08	11.09	11.09
V3	0.56	0.54	0.58	0.47	0.97	0.94	1.01	1.00	27.07	47.04	21.35	21.53	13.07	22.06	11.08	11.07
V4	0.48	0.43	0.52	0.41	0.96	0.90	1.00	0.99	28.17	45.85	21.96	22.45	13.06	22.05	11.06	11.06
V5	0.42	0.44	0.59	0.36	0.94	0.86	1.00	0.98	26.95	44.35	21.95	17.14	13.05	22.05	11.05	11.68
V6	0.38	0.39	0.66	0.33	0.93	0.81	0.99	0.96	28.93	45.49	22.46	24.30	13.03	22.05	11.04	11.02
Avg	0.63	0.63	0.63	0.50	0.97	0.91	1.01	0.99	26.57	45.67	21.56	21.19	13.07	22.06	11.07	11.17
String Stability Ratio: 0.9345/0.8074/0.9922/0.9628 Result: STABLE/STABLE/STABLE/STABLE																
S2. Emergency Platooning																
V1	4.76	5.16	12.96	2.78	3.04	2.98	4.89	3.00	5.66	10.35	2.78	9.25	10.52	32.05	8.35	17.17
V2	6.71	2.46	18.43	2.28	2.75	2.94	7.18	3.00	collision	19.51	2.28	8.38	34.21	30.51	8.24	16.36
V3	6.42	1.40	18.40	1.93	2.75	2.89	7.63	2.96	0.55	20.10	2.02	9.42	11.06	30.51	8.26	12.21
V4	6.24	1.49	23.45	1.58	2.75	2.86	7.85	2.93	collision	19.46	collision	10.60	11.18	30.65	9.39	12.19
Avg	6.03	2.63	18.31	2.14	2.82	2.92	6.89	2.97	collision	17.35	collision	9.41	16.74	30.93	8.56	14.48
String Stability Ratio: collision/0.9526/collision/0.9751 Result: collision/STABLE/collision/STABLE																
S3. Truck Platooning																
V1	1.58	2.05	2.00	2.10	2.10	2.08	2.14	2.02	16.85	26.54	12.53	12.74	16.22	25.16	14.26	14.20
V2	1.37	1.30	1.54	1.60	2.13	1.99	2.11	2.01	17.50	26.53	13.10	13.26	11.35	25.11	9.34	9.34
V3	1.25	0.85	1.43	1.34	2.15	1.87	2.09	2.00	17.28	27.41	13.25	13.36	11.34	25.05	9.33	9.32
V4	1.19	0.65	1.34	1.20	2.17	1.85	2.06	1.98	17.09	26.29	13.36	13.31	11.32	25.05	9.31	9.30
V5	1.18	0.56	1.81	1.08	2.19	1.80	2.04	1.95	16.89	25.24	13.49	14.53	11.30	25.04	9.30	9.29
V6	1.18	0.57	2.85	0.98	2.21	1.74	2.03	1.92	16.73	24.22	13.72	14.78	11.28	25.03	9.28	9.27
Avg	1.29	1.00	1.83	1.38	2.16	1.89	2.08	1.98	17.06	26.04	13.24	13.66	12.14	25.07	10.14	10.12
String Stability Ratio: 1.1041/0.8703/1.0130/0.9604 Result: UNSTABLE/STABLE/UNSTABLE/STABLE																

**Table 9:** Sensitivity Analysis of Safe PF-CACC to the LPF Time Constant in S2.

$\tau_{f}$ (s)	Max Jerk (m/s^3^)	Min TTC (s)	Mean Spacing (m)
0.0	3.58	7.71	14.71
0.5	2.78	7.70	14.71
1.0	2.78	7.83	14.70
2.0	2.78	8.38	14.48
3.0	2.78	8.37	14.67

## Data Availability

Data are contained within the article.
